# Understanding the impact of preprocessing pipelines on neuroimaging cortical surface analyses

**DOI:** 10.1093/gigascience/giaa155

**Published:** 2021-01-22

**Authors:** Nikhil Bhagwat, Amadou Barry, Erin W Dickie, Shawn T Brown, Gabriel A Devenyi, Koji Hatano, Elizabeth DuPre, Alain Dagher, Mallar Chakravarty, Celia M T Greenwood, Bratislav Misic, David N Kennedy, Jean-Baptiste Poline

**Affiliations:** Montreal Neurological Institute & Hospital, McGill University, Neurology and Neurosurgery, 3801 University Street, Montreal, H3A 2B4H3A 2B4, Montreal, QC, Canada; Lady Davis Institute for Medical Research, McGill University, Montreal, QC, Canada; Kimel Family Translational Imaging-Genetics Research Lab, Centre for Addiction and Mental Health, Toronto, ON, Canada; Montreal Neurological Institute & Hospital, McGill University, Neurology and Neurosurgery, 3801 University Street, Montreal, H3A 2B4H3A 2B4, Montreal, QC, Canada; Computational Brain Anatomy Laboratory, Douglas Mental Health Institute, Verdun, QC, Canada; Department of Psychiatry, McGill University, Montreal, QC, Canada; Montreal Neurological Institute & Hospital, McGill University, Neurology and Neurosurgery, 3801 University Street, Montreal, H3A 2B4H3A 2B4, Montreal, QC, Canada; Montreal Neurological Institute & Hospital, McGill University, Neurology and Neurosurgery, 3801 University Street, Montreal, H3A 2B4H3A 2B4, Montreal, QC, Canada; Montreal Neurological Institute & Hospital, McGill University, Neurology and Neurosurgery, 3801 University Street, Montreal, H3A 2B4H3A 2B4, Montreal, QC, Canada; Computational Brain Anatomy Laboratory, Douglas Mental Health Institute, Verdun, QC, Canada; Department of Psychiatry, McGill University, Montreal, QC, Canada; Department of Biomedical Engineering, McGill University, Montreal, QC, Canada; Lady Davis Institute for Medical Research, McGill University, Montreal, QC, Canada; Ludmer Centre for Neuroinformatics & Mental Health, McGill University, Montreal, QC, Canada; Gerald Bronfman Department of Oncology; Department of Epidemiology, Biostatistics & Occupational Health Department of Human Genetics, McGill University, Montreal, QC, Canada; Montreal Neurological Institute & Hospital, McGill University, Neurology and Neurosurgery, 3801 University Street, Montreal, H3A 2B4H3A 2B4, Montreal, QC, Canada; Child and Adolescent Neurodevelopment Initiative, University of Massachusetts, Worcester, MA, USA; Montreal Neurological Institute & Hospital, McGill University, Neurology and Neurosurgery, 3801 University Street, Montreal, H3A 2B4H3A 2B4, Montreal, QC, Canada; Ludmer Centre for Neuroinformatics & Mental Health, McGill University, Montreal, QC, Canada

**Keywords:** neuroimaging, reproducibility, cortical thickness, preprocessing pipelines

## Abstract

**Background:**

The choice of preprocessing pipeline introduces variability in neuroimaging analyses that affects the reproducibility of scientific findings. Features derived from structural and functional MRI data are sensitive to the algorithmic or parametric differences of preprocessing tasks, such as image normalization, registration, and segmentation to name a few. Therefore it is critical to understand and potentially mitigate the cumulative biases of pipelines in order to distinguish biological effects from methodological variance.

**Methods:**

Here we use an open structural MRI dataset (ABIDE), supplemented with the Human Connectome Project, to highlight the impact of pipeline selection on cortical thickness measures. Specifically, we investigate the effect of (i) software tool (e.g., ANTS, CIVET, FreeSurfer), (ii) cortical parcellation (Desikan-Killiany-Tourville, Destrieux, Glasser), and (iii) quality control procedure (manual, automatic). We divide our statistical analyses by (i) method type, i.e., task-free (unsupervised) versus task-driven (supervised); and (ii) inference objective, i.e., neurobiological group differences versus individual prediction.

**Results:**

Results show that software, parcellation, and quality control significantly affect task-driven neurobiological inference. Additionally, software selection strongly affects neurobiological (i.e. group) and individual task-free analyses, and quality control alters the performance for the individual-centric prediction tasks.

**Conclusions:**

This comparative performance evaluation partially explains the source of inconsistencies in neuroimaging findings. Furthermore, it underscores the need for more rigorous scientific workflows and accessible informatics resources to replicate and compare preprocessing pipelines to address the compounding problem of reproducibility in the age of large-scale, data-driven computational neuroscience.

## Introduction

Reproducibility, a presumed requisite of any scientific experiment, has recently been under scrutiny in the field of computational neuroscience [1–7]. Specifically, the replicability and generalizability of several neuroimaging pipelines and the subsequent statistical analyses have been questioned, potentially owing to insufficient sample size [8], imprecise or flexible methodological and statistical a priori assumptions [9–11], and poor data/code-sharing practices [12, 13]. Broadly speaking, reproducibility can be divided into 2 computational goals [14]. The first goal is replicability, which implies that a re-executed analysis on the identical data should always yield the same results. The second goal pertains to generalizability, which is assessed by comparing the scientific findings under variations of data and analytic methods. Typically, findings are deemed generalizable when similar (yet independent) data and analysis consistently support the experimental hypothesis. This in turn raises the issue of defining what constitutes “similar” data and analytic methodology. Nonetheless, traditionally experimental validation on independent datasets has been utilized to assess generalizability. However, as the use of complex computational pipelines has become an integral part of modern neuroimaging analysis [15], comparative assessment of these pipelines and their impact on the generalizability of findings deserves more attention.

We present a comparative assessment of multiple structural neuroimaging preprocessing pipelines on the Autism Brain Imaging Data Exchange (ABIDE), a publicly accessible dataset comprising healthy controls and individuals with autism spectrum disorder (ASD) [16]. A few studies have previously highlighted the variability in neuroimaging analyses introduced by the choice of a preprocessing pipeline for structural MR images [17, 18]; however, they have not focused on the relative impact of analysis tools, quality control (QC), and parcellations on the consistency of results. The inconsistencies in the results arise from several algorithmic and parametric differences that exist in the preprocessing tasks, such as image normalization, registration, and segmentation within pipelines. It is critical to understand and mitigate the cumulative biases of the pipelines to disambiguate biological effect from methodological variance. We further replicate our findings on the Human Connectome Project (HCP) data.

**Figure 1:**
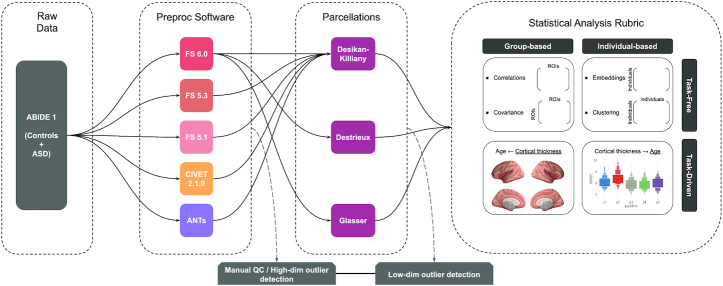
MR image-preprocessing pipeline building blocks. Each block comprises several potential choices for a typical structural MR image analysis. Only a subset of the possible pipeline variations is analyzed, as shown with arrows. Manual quality control and automatic outlier detection are shown as auxiliary tasks, which can be performed at various stages.

For this purpose, we propose a comprehensive investigation of the impact of pipeline selection on cortical thickness measures—a widely used (3,129 hits on PubMed and 42,200 hits on Google Scholar for “cortical thickness” AND “magnetic resonance imaging” search query), fundamental phenotype—and its statistical association with biological age. We limit the scope of pipeline variation to 3 axes of parameter selection: (i) image-processing tool, (ii) anatomical priors, and (iii) QC (see Fig. 1). The effect of the variation is measured on 2 types of statistical analyses, namely, (i) neurobiological inference carried out using general linear modelling (GLM) techniques at the group level and (ii) individual predictions from machine learning (ML) models. We note that here the focus is on the preprocessing stages of a computational pipeline, and the effect of dataset and statistical model selection is thus outside of the present scope. Our goal is not to explain potential differences in results or establish criteria to rank pipelines or tools but to document the pipeline effect and provide best-practice recommendations to the neuroscience community with respect to pipeline variation, also referred to as pipeline vibration effects.

Although here we do not focus on identifying biological differences between ASD case and control groups, we use the case-control samples to gain insight into the effect of diagnosis on reproducibility analysis—which is a critical evaluation for clinical applications. Additionally, we use a data sample from the HCP as a validation dataset (Van Essen et al. [19]) to assess whether our findings can be replicated on an independent dataset. Note that the scope of this secondary analysis is limited to a proof-of-concept dataset comparison.

We organize our comparative assessments on the ABIDE dataset as follows. We report comparisons across the 3 aforementioned axes of variation. This comprises 5 neuroimaging preprocessing tools: (i) FreeSurfer (FS) 5.1, (ii) FS 5.3, (iii) FS 6.0, (iv) CIVET 2.1.0, and (v) ANTS; 3 anatomical priors (i.e., cortical parcellations): (i) Desikan-Killiany-Tourville (DKT), (ii) Destrieux, and (iii) Glasser; and 5 QC procedures: (i) no QC, (ii) manual lenient, (iii) manual stringent, (iv) low-dimensional automatic outlier detection (i.e., <500 regions of interest [ROIs]), and (v) high-dimensional automatic outlier detection (i.e., >100,000 vertices). The entire combinatorial set of comparisons (5 software × 3 parcellations × 5 QC) is not feasible owing to practical limitations (described later), and therefore we report results for 5 tool procedures and 3 atlases across 5 QC procedures (5 software + 3 parcellations) × 5 QC, as shown by the connecting arrows in Fig. 1. We use these 40 variations of preprocessed data with 4 types of statistical analyses based on a method type (i.e., task-free vs task-driven) and an inference objective (neurobiological vs individual), as described in detail in the Materials and Methods section.

## Materials and Methods

### Participants

**Figure 2:**
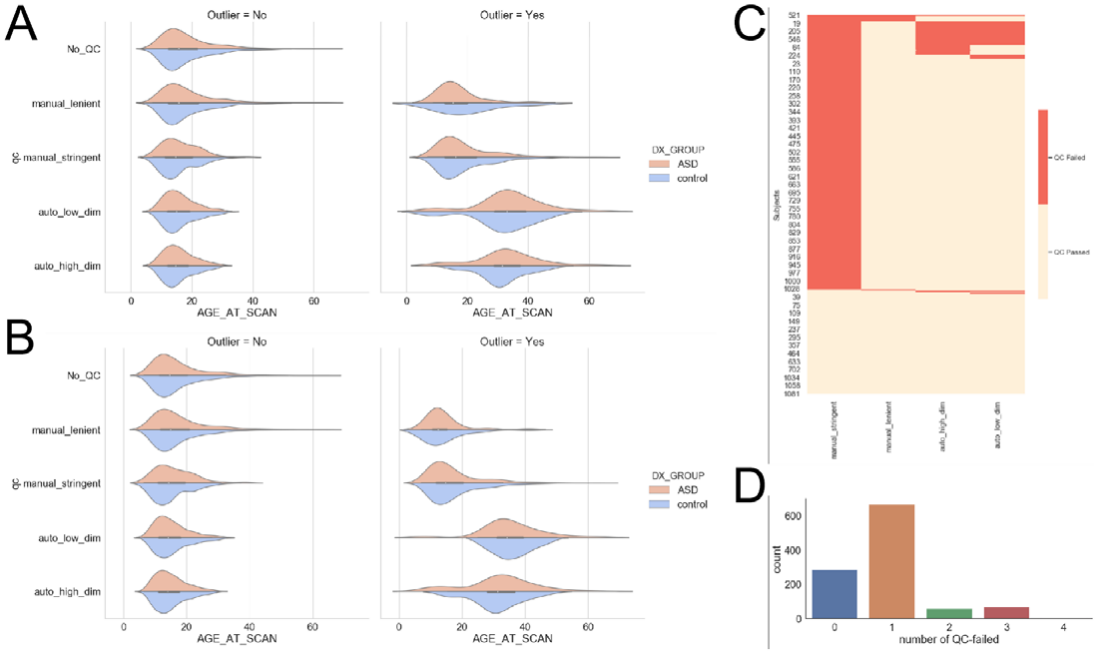
(A) Age distributions for sample subsets used for software comparison analysis. (B) Age distributions for sample subsets used for parcellation comparison analysis. See Table 1 for sample sizes. (C) Failed QC participants overlap across the four (manual and automatic outlier detection) procedures. (D) Distribution of total failures across the four QC (manual and automatic outlier detection) procedures. This shows that most data failed QC by at least one QC procedure, but only a small number were rejected by two or three QC procedures.

Participants from the ABIDE dataset were used for this study [16]. The ABIDE 1 dataset comprises 573 controls and 539 individuals with autism spectrum disorder (ASD) from 16 international sites. The neuroimaging data of these individuals were obtained from the ABIDE preprocessing project [20], the Neuroimaging Tools and Resources Collaboratory (NITRC) [21], and the DataLad repository [22]. Different subsets of individuals were used for various analyses based on (i) specific image-processing failures, (ii) need for a common sample set for software tool comparison, and (iii) QC procedures. The demographic description of these subsets is provided in Table 1 and Fig. 2. The complete lists of participants can be obtained from the code repository [23].

**Table 1:** Participant demographic characteristics for different analyses

Comparisons	QC	Diagnosis	Number of Participants	Age: mean (SD)	Sex (M/F)
Software tools	No QC (N = 778)	Controls	415	17.8 (7.7)	346/69
		ASD	363	18.3 (8.7)	320/43
	Lenient manual (N = 748)	Controls	407	17.8 (7.6)	338/69
		ASD	341	18.4 (8.8)	300/41
	Stringent manual (N = 194)	Control	113	15.6 (5.5)	93/20
		ASD	81	16.2 (5.8)	71/10
	Auto QC low-dimensional (N = 683)	Controls	371	16.2 (5.4)	309/62
		ASD	312	15.9 (5.0)	276/36
	Auto QC high-dimensional (N = 662)	Controls	356	15.6 (5.0)	293/63
		ASD	306	15.7 (4.9)	269/37
Parcellations	No QC (N = 1,047)	Controls	552	17.0 (7.5)	456/96
		ASD	495	17.1 (8.4)	436/59
	Lenient manual (N = 975)	Controls	525	17.1 (7.5)	430/95
		ASD	450	17.4 (8.6)	395/55
	Stringent manual (N = 240)	Controls	137	15.0 (5.6)	112/25
		ASD	103	16.1 (6.3)	91/12
	Auto QC low-dimensional (N = 961)	Controls	516	15.6 (5.6)	422/94
		ASD	445	15.0 (5.1)	390/55
	Auto QC high-dimensional (N = 912)	Controls	483	15.0 (4.9)	393/90
		ASD	429	14.9 (4.9)	377/52

The subsets of individuals are based on (i) specific image-processing failures, (ii) need for a common sample set for software tool comparison, and (iii) quality control (QC) procedures.

### MRI processing and cortical thickness measurements

#### FreeSurfer

FS delineates the cortical surface from a given MRI scan and quantifies thickness measurements on this surface for each brain hemisphere [24, 25]. The default pipeline consists of (i) affine registration to the MNI305 space [26]; (ii) bias field correction; (iii) removal of skull, cerebellum, and brainstem regions from the MR image; (iv) estimation of white matter (WM) surface based on MR image intensity gradients between the WM and grey matter (GM); and (v) estimation of pial surface based on intensity gradients between the GM and cerebrospinal fluid. The distance between the white and pial surfaces provides the thickness estimate at a given location of cortex. For detailed description refer to [27]. The individual cortical surfaces are then projected onto a common space (i.e., fsaverage) characterized by 163,842 vertices per hemisphere to establish interindividual correspondence.

In this work, the cortical thickness for each MR image was computed using FS 5.1, 5.3, and 6.0 versions. The FS5.1 measurements were obtained from the ABIDE preprocessing project [20]. The standard recon-all pipeline with “-qcache” flag was used to process and resample the images onto common (fsaverage) space. The FS5.3 measurements were extracted using the standard ENIGMA cortical thickness pipeline [28]. Last, the FS6.0 measurements were obtained using the standard recon-all pipeline with -qcache flag as well. Compute Canada [29] and CBRAIN [30] computing infrastructures were used for processing of FS5.3 and FS6.0 data.

#### CIVET

CIVET 2.1 [31] preprocessing was performed on the data obtained from NITRC. The standard CIVET pipeline consists of (i) N3 bias correction [32]; (ii) affine registration to the MNI ICBM 152 stereotaxic space; (iii) tissue classification into WM, GM, and cerebrospinal fluid; (iv) brain splitting into left and right hemispheres for independent surface extraction; and (v) estimation of WM, pial, and GM surfaces. The cortical thickness is then computed using the distance (i.e., Tlink metric) between WM and GM surfaces at 40,962 vertices per hemisphere.

#### ANTS

The MRI dataset preprocessed with ANTS (ANTS - Advanced Normalization ToolS, RRID:SCR_004757) version May-2017 was obtained from the ABIDE preprocessing project [20]. The detailed description of the ANTS cortical thickness pipeline can be found here [17]. Briefly, the ANTS pipeline consists of (i) N4 bias correction [33], (ii) brain extraction, (iii) prior-based segmentation and tissue-based bias correction, and (iv) diffeomorphic registration-based cortical thickness estimation [34]. One key differentiating aspect of ANTS is that it uses quantification of cortical thickness in the voxel-space, unlike FS or CIVET, which operate with vertex-meshes.

### Cortical parcellations

The regions of interest (ROIs) were derived using 3 commonly used cortical parcellations, namely, (i) DKT [35], (ii) Destrieux [36], and (iii) Glasser [37]. DKT parcellation consists of 31 ROIs per hemisphere and is a modification of the Desikan–Killiany protocol [38] to improve cortical labeling consistency. DKT label definitions are included in all 3 FS, CIVET, and ANTS pipelines, which allows the comparison of cortical phenotypic measures across these tools. The Destrieux parcellation is a more detailed anatomical parcellation proposed for a precise definition of cortical gyri and sulci. The Destrieux parcellation comprises 74 ROIs per hemisphere and is also available in the FS pipeline. In contrast to these structural approaches, the Glasser parcellation was created using multimodal MR acquisitions from 210 HCP participants [39] with 180 ROIs per hemisphere. Glasser label definitions are available in the “fsaverage” space [40], i.e., the common reference space used by FS, allowing comparisons across multiple parcellations.

### Quality control

We used manual (i.e., visual) and automatic (statistical outlier detection) procedures to investigate the effect of QC on thickness distributions derived from combinations of the different software tools and cortical parcellations. The manual QC checks were performed on the extracted cortical surfaces by 2 independent expert raters [41, 42]. The 2 raters used different criteria for assessing the quality of surface delineation. This in turn yielded 2 lists of QC-passed participants from “lenient” and “stringent” criteria. We note that these lenient and stringent QC lists were generated independently using FS and CIVET images, respectively, and then applied to all pipeline variations. The automatic QC was performed using an outlier detection algorithm based on a random min-max multiple deletion (RMMMD) procedure (Barry et al. in preparation). The RMMMD algorithm is a high-dimensional extension of the Cook influence measure to identify influential observations. The outlier detection method was applied separately to high-dimensional vertex-wise output and low-dimensional aggregate output based on cortical parcellations for each software and parcellation choice.

### Statistical analysis

We categorize the downstream statistical analyses into a 2 × 2 design. The first factor consists of either (i) unsupervised, task-free (TF) analyses or (ii) supervised, task-driven (TD) analyses. The second factor corresponds to either (i) neurobiological (N) tasks investigating the biological effect across groups of individuals or (ii) individual (I) tasks predicting individual-specific states (see Table 2). The task-free, neurobiologically oriented analyses (TF-N) aim at quantifying similarity of preprocessed features (i.e., ROI-wise cortical thickness values) without the explicit constraint of an objective function. Task-driven, neurobiologically oriented analyses (TD-N) quantify feature similarity in the context of a GLM framework. Individually oriented analyses formulate the duality of neurobiological analyses, with a focus on individual similarity in task-free (TF-I) and task-driven (TD-I) contexts.

**Table 2:** Types of analysis performed for each axis of variation

Axis of variation	Analysis type	Neurobiology (N)	Individual (I)
Software Tool	Task-Free (TF)	Feature correlations and covariance	Individual embeddings and clustering
	Task-Driven (TD)	ROI ~Age + coviariates	Age ← ROIs + coviariates
			
Cortical Parcellation	Task-Free (TF)	N/A	N/A
	Task-Driven (TD)	ROI ~Age + coviariates	Age ← ROIs + coviariates
			
Quality control	Task-Free (TF)	N/A	N/A
	Task-Driven (TD)	ROI ~Age + coviariates	Age ← ROIs + coviariates

N/A: not applicable.

Previous work has reported varying degrees of association and predictability of age from cortical thickness measures in neurotypical and ASD cohorts [43–47]. We therefore selected biological age as our objective for the TD analyses. Although other clinical variables (e.g., diagnosis) could be used, the availability and unambiguity of age quantification across datasets simplifies comparison of the different analyses.

For TF-N analysis we evaluate the pairwise correlation and covariance of features using the Pearson *r* metric. For TF-I analysis, we assess individual similarity using t-distributed stochastic neighbour embedding (t-SNE) and hierarchical clustering with Euclidean distance and Ward linkage metrics. For TD-N analysis, we build a GLM to associate cortical thickness and biological age with sex and data collection site as covariates. For TD-I analysis, we train a random forest (RF) model for age prediction using cortical thickness, sex, and data collection site as predictors. Of note, we also assess the importance assigned to cortical features by the RF model. ML model performance and feature importance is assessed within 100 iterations of a shuffle-split cross-validation paradigm.

We also note that not all pipeline variations can be assessed easily within this to 2 × 2 statistical analyses design. As mentioned before we only analyze a subset [(5 + 3) × 5] of possible pipeline variations, and compare the 5 software tools using common DKT parcellation. Tool comparison with Destrieux and Glasser parcellations is not trivial owing to their unavailability for CIVET and ANTS. This also limits our comparison across 3 parcellations solely with FS 6.0. We do however compare all 5 QC procedures with these combinations. The analyses performed in this work are provided in Table 2. The code used for the analyses is available at [23].

### Validation study

The T1w images of 1,108 individuals from the HCP dataset [19] were successfully preprocessed using FS 6.0 and CIVET 2.1, respectively, and mean cortical thickness measurements in the DKT ROIs were obtained. Identical to the ABIDE analysis, we evaluated the pairwise correlations and covariance of features between CIVET 2.1 and FS 6.0 using the Pearson *r* metric, then we compared it using the same approach as for the ABIDE dataset.

## Results

### Task-free neurobiological (TF-N) analysis

**Figure 3:**
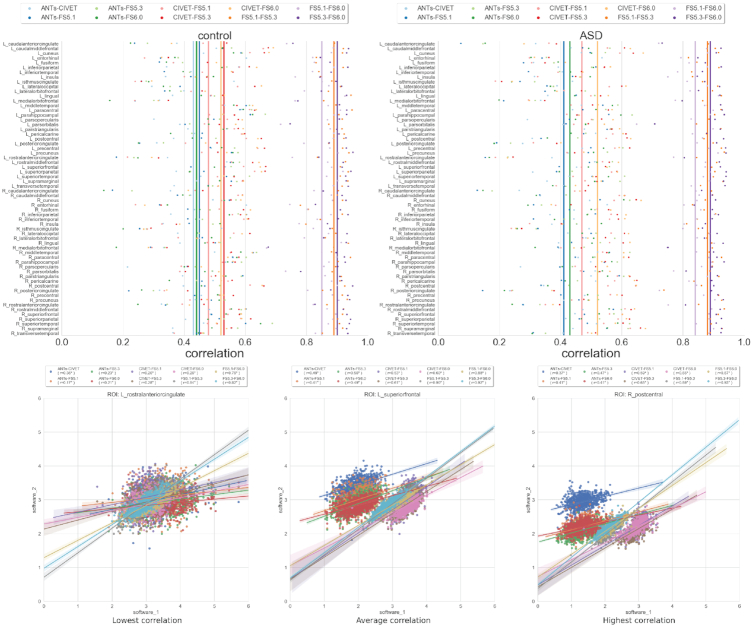
Task-free neurobiological (TF-N) analysis. (Top) Correlation between cortical thickness values for software pairs measured independently over ROIs for control and ASD groups. The vertical lines represent the mean correlation across all ROIs. The ROIs are defined using Desikan-Killiany-Tourville (DKT) parcellation. (Bottom) Distribution of cortical thickness values of exemplar ROIs with lowest, average, and highest median correlation across software pairs.

Feature comparisons across the 5 software tools are performed using common DKT parcellation. The pairwise comparisons between software tools are performed on the basis of the ROI-wise Pearson correlations between thickness measures produced by each tool (see Fig. 3, Table 3). The pairwise comparisons between FS, CIVET, and ANTS tools show very little similarity, with correlation values averaged over all regions remaining low (*r*ϵ[0.39, 0.52]). The comparisons between different versions of FS show relatively better average correlation performance (*r*ϵ[0.83, 0.89]). Stratifying comparisons by diagnosis does not improve correlation. ROI-specific performance shows the lowest median correlation for the left rostral-anterior-cingulate (*r* = 0.27) and left and right isthmus-cingulate (*r* = 0.29,0.31) regions and the highest median correlation for the left cuneus (*r* = 0.63), right postcentral (*r* = 0.63), and left caudal-middle-frontal (*r* = 0.62) regions across all software pairs. The pairwise thickness distributions for 3 randomly selected exemplar ROIs corresponding to different levels of median correlations across software tools are shown in Fig. 3. The exemplar ROI comparison suggests that ROIs with high correlation levels tend to have lower overlap between the pairwise thickness distributions.

**Table 3:** Mean ROI correlations between software pairs for control and ASD cohorts

	Controls					ASD				
	ANTS	CIVET	FS5.1	FS5.3	FS6.0	ANTS	CIVET	FS5.1	FS5.3	FS6.0
ANTS	1	0.43	0.45	0.48	0.44	1	0.39	0.39	0.46	0.41
CIVET		1	0.48	0.52	0.52		1	0.44	0.48	0.49
FS5.1			1	0.89	0.84			1	0.87	0.83
FS5.3				1	0.89				1	0.88
FS6.0					1					1

**Figure 4:**
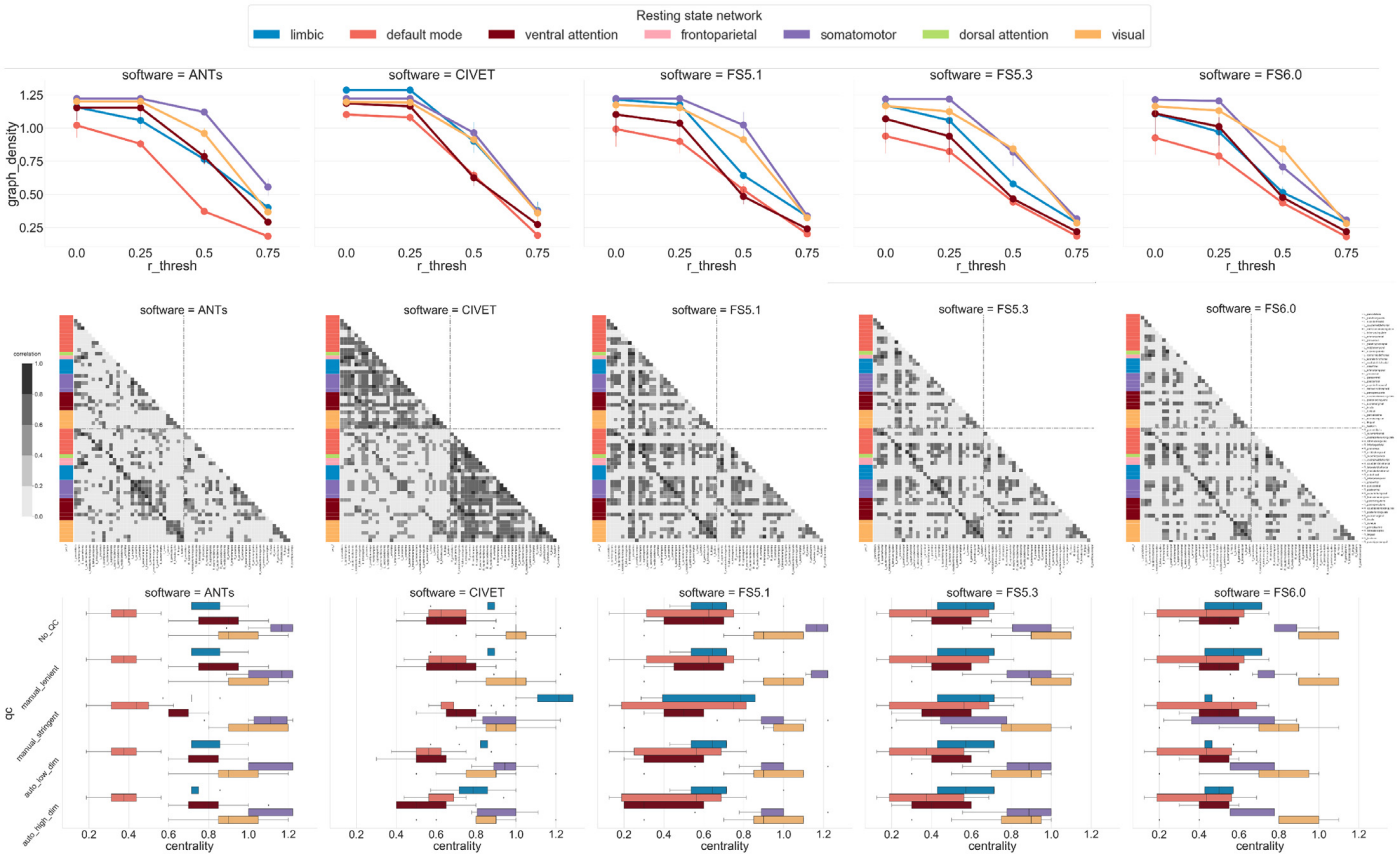
Task-free neurobiological (TF-N) analysis. (Top) Graph density for different correlation cut-off thresholds used for constructing a structural network. The error bars show variation due to the QC procedures. (Middle) Structural covariance of each software tool measured as inter-ROI correlation with cut-off value of 0.5. For simplicity, the covariance plot is generated with original data. The covariance patterns are grouped on the basis of Yeo resting state networks membership. (Bottom) Distribution of regional degree-centrality metric per Yeo network for each software with different QC procedures. Note that frontoparietal and dorsal attentional networks are excluded from some analyses owing to the small number of DKT ROIs in these networks.

The covariance matrix of ROIs and subsequently derived structural network metrics reveal several software-specific differences. First, the covariance matrix shows large variation of patterns across software tools (see Fig. 4 [middle]). All software tools show strong bilateral symmetry evidenced by the high correlation values on the diagonal representing hemispheric ROI pairs. Interestingly, CIVET features show stronger intrahemispheric correlation between ROIs compared to the interhemispheric values. The DKT ROIs are grouped on the basis of their membership in the Yeo resting state networks [48] to compute graph theoretic metrics. Figure 4 shows the variation in the 2 commonly used metrics. Figure 4 (top) shows the impact of correlation threshold, typically used for denoising graph edges, on the fundamental measure of graph density. The 3 FS versions show relatively similar performance for all resting state networks, with somatomotor and default mode exhibiting highest and lowest densities, respectively. Compared to FS values, ANTS and CIVET show different magnitudes and/or rankings of graph densities across networks. These differences are further amplified in the graph degree-centrality measurements across networks. Figure 4 (bottom) shows high intranetwork regional variance in degree-centrality for FS versions. This variance is relatively smaller for ANTS and CIVET, but these software tools generally showdifferent magnitudes of centrality, particularly in limbic and default mode networks.

Comparison across QC procedures did not show any substantial effect on correlation values. Feature comparison for a given software tool (e.g., FS6.0) across different parcellations is not trivial owing to the lack of correspondence between various parcellation schemes.

### Task-free individual (TF-I) analysis

**Figure 5:**
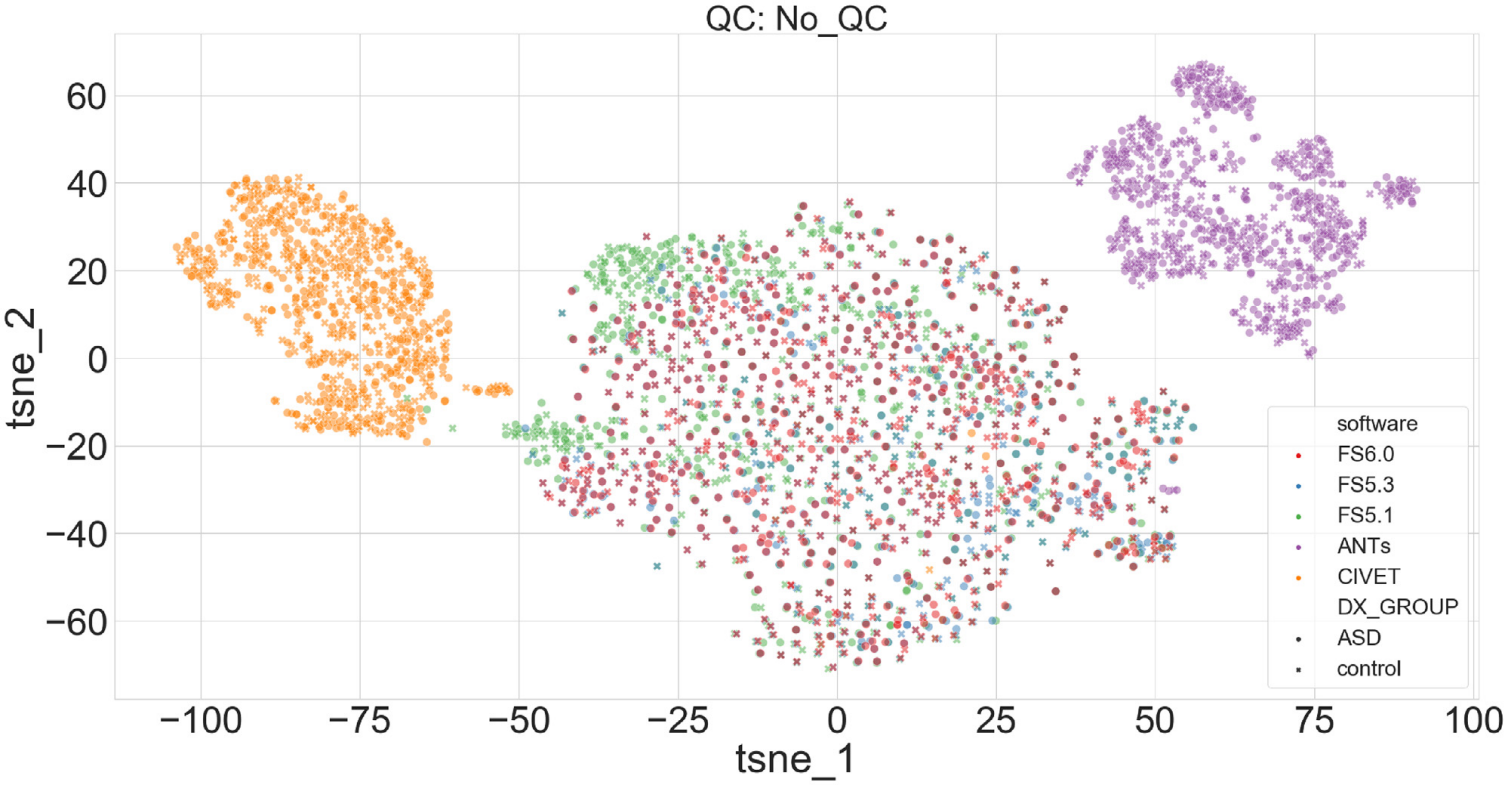
Task-free individual (TF-I) analysis. 2D t-SNE representation of all individuals (no QC). The colours indicate the software tool used, and the marker style, the diagnostic group.

Individual comparisons using thickness measures from DKT parcellation are performed across the 5 software tools with an identical set of participants. Commonly used 2D t-SNE embeddings show strong similarity between participants for a given software tool (see Fig. 5). The 3 FS versions are much more similar to each other than any FS version is to CIVET or ANTS, reflecting that the different versions of FS share methodological and technical components. Individual covariance is quantified using clustering consistency (CC), which measures the fraction of pairs of individuals assigned to the same cluster with 2 different feature sets (e.g., ANTS vs CIVET). Based on the CC metric, hierarchical clustering with Euclidean distance similarity and Ward linkage criterion shows poor stability (CC$\in$[0.52, 0.61]) across software tools and between FS versions (see Table 4). In contrast, hierarchical clustering with correlation metric and average linkage criterion shows highly stable cluster membership (CC$\in$[0.962, 0.997]).

**Table 4:** Clustering consistency between software pairs

	Similarity: Euclidean distance, linkage: Ward method					Similarity: correlation, linkage: average				
	ANTS	CIVET	FS5.1	FS5.3	FS6.0	ANTS	CIVET	FS5.1	FS5.3	FS6.0
ANTS	0.797	0.5	0.521	0.517	0.522	0.991	0.970	0.962	0.972	0.972
CIVET		0.717	0.5	0.5	0.5		0.994	0.982	0.992	0.992
FS5.1			0.78	0.609	0.529			0.997	0.990	0.985
FS5.3				0.703	0.499				0.997	0.995
FS6.0					0.619					0.997

The diagonal shows expected overlap based on 100 bootstrap samplings of features (31 ROIs) for a given software tool.

Comparison across QC procedures did not show any substantial impact on t-SNE representations or CC values. Individual comparisons across different parcellations for a given software tool (e.g., FS6.0) are not particularly informative owing to the lack of correspondence between various parcellation spaces.

### Task-driven neurobiological (TD-N) analysis

**Figure 6:**
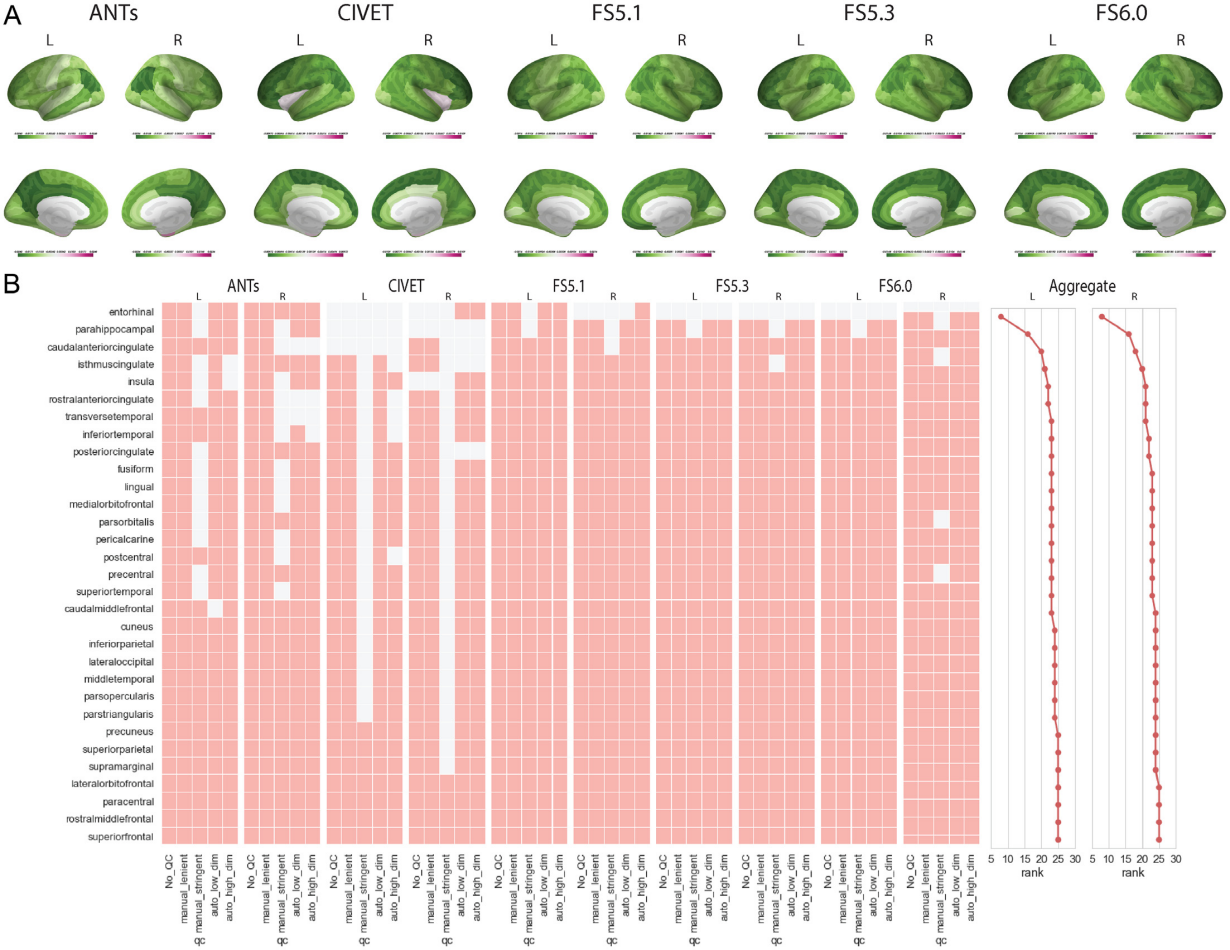
Task-driven neurobiological (TD-N) analysis. (A) GLM β maps for each software tool with the “no QC" sample. Apart from Enenrhinal cortex and insular regions (CIVET), cortical thickness at all other cortical regions is negatively associated with age. (B) Comparison of significant ROIs with various software a toolsnd QC levels. T Aolored square implies r that theegion was significant. Significance levels are corrected for multiple comparisons using the Bonferonri procedure. The last panel (Aggregate) shows ROI rank based on performance agreement among 5 software antools d 5 QC procedures (row sums).

The mass-univariate regression models per ROI region suggest cortex-wide association between age and thickness values for all software tools, with the exception of the CIVET-based analysis, which excludes bilateral insular regions (see Fig. 6). QC procedures seem to have varying impact on the significant regions depending on the software tool. The aggregate ranking suggests higher variation in significant regions for ANTS and CIVET. In contrast the FS versions offer relatively similar performance—with consistent exclusion of entorhinal regions. The stringent manual QC sample severely reduces the number of significant regions, which may be due to reduced statistical power.

**Figure 7:**
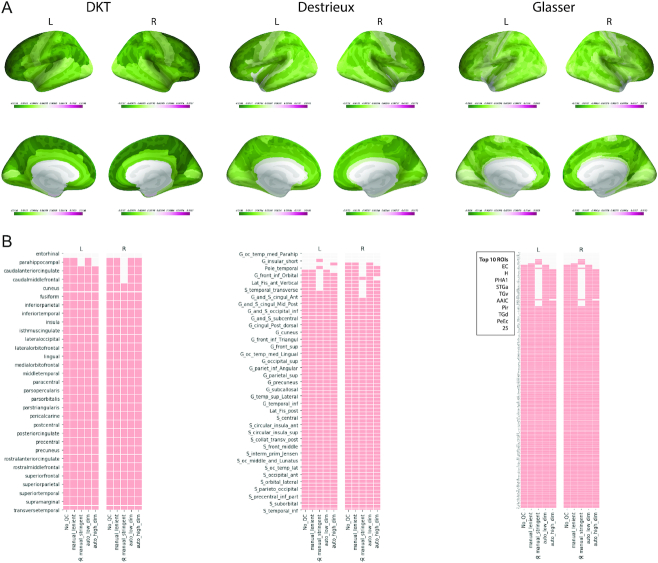
Task-driven neurobiological (TD-N) analysis. (A) GLM β maps for each parcellation with the “no QC" sample. (B) Comparison of significant ROIs with various parcellations and QC levels. A coloured square implies that the region was significant. Significance levels are corrected for multiple comparisons using the Bonferroni procedure.

Parcellation comparisons for FS 6.0 reaffirm cortex-wide association between age and thickness values across the 3 parcellation schemes, with some exclusions in the medial and superior temporal gyri for Destrieux and in entorhinal cortex (EC), superior temporal gyrus region a (STGa), piriform cortex (PIR), TGd, TGv, PHA1, and perirhinal ectorhinal cortex (PeEC) for Glasser (see Fig. 7). Lenient QC does not seem to change the distribution of significant regions. However, results based on stringent and automatic QC additionally exclude regions from precentral gyri for all 3 atlases.

### Task-driven individual (TD-I) analysis

**Figure 8:**
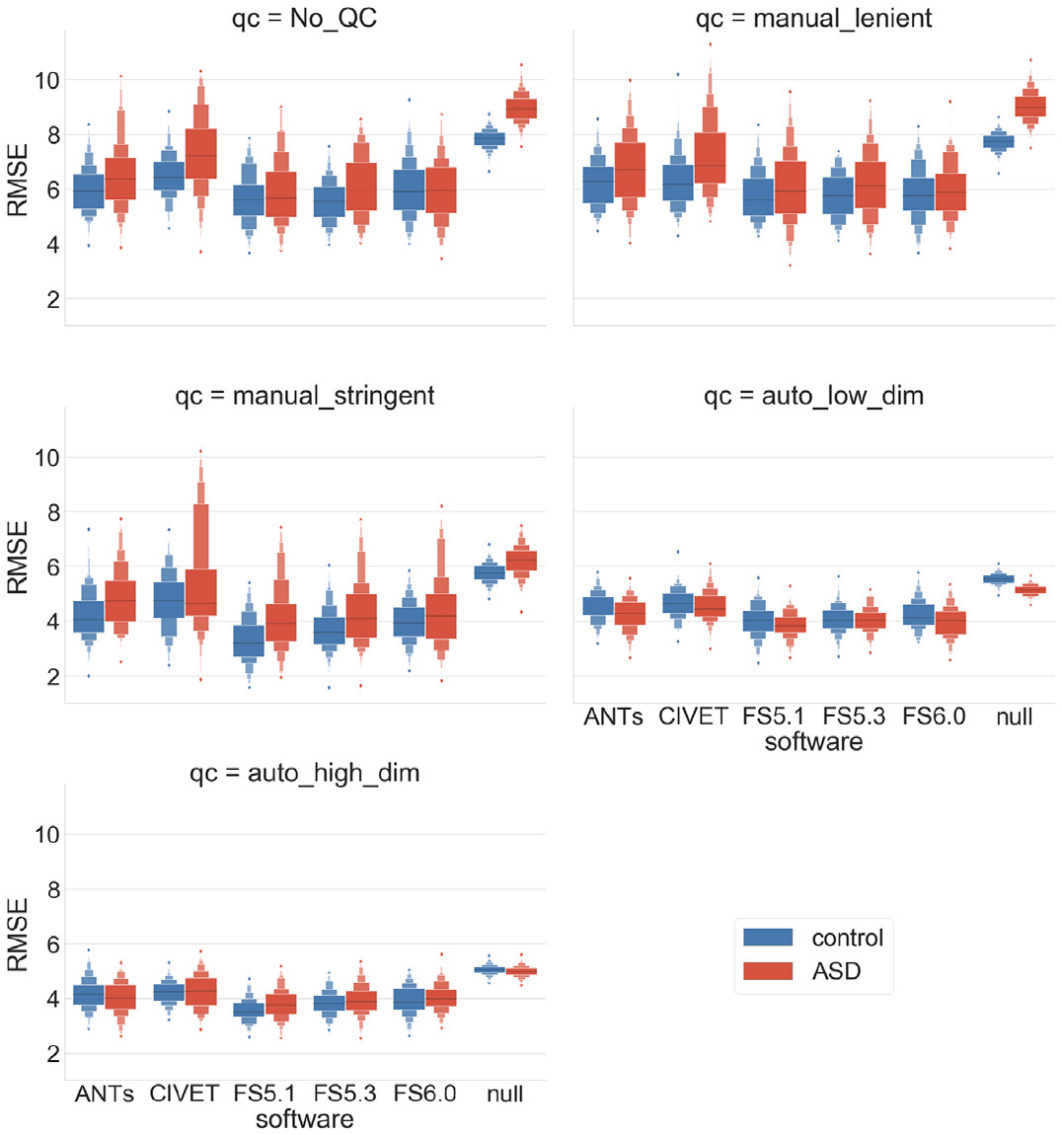
Task-driven individual (TD-I) analysis. Individual age prediction with various software tools and QC levels stratified by diagnosis. Performance (measured by Root Mean Square Error, RMSE) is cross-validated using a random forest model over 100 shuffle-split iterations.

The RF model–based predictions show consistent root mean square error (RMSE) performance (5.7–7.2 years) across software tools, with FS versions showing marginally lower error (see Fig. 8). All model performances are statistically significant when compared against a null model. The mean RMSE for the control cohort is lower than for the ASD cohort; as expected per the null model, however, the difference is statistically insignificant. Lenient QC does not have an effect on RMSE distributions. Stringent QC reduces the mean RMSE for all software tools (3–5 years) and the null model. Automatic QC reduces the average RMSE, as well as its variance, for all software tools (3.8–4.7 years). Interestingly with the automatic QCs (low- and high-dimensional), the null models' expectations are reversed because the mean RMSE for ASD participants is now lower than that of controls.

**Figure 9:**
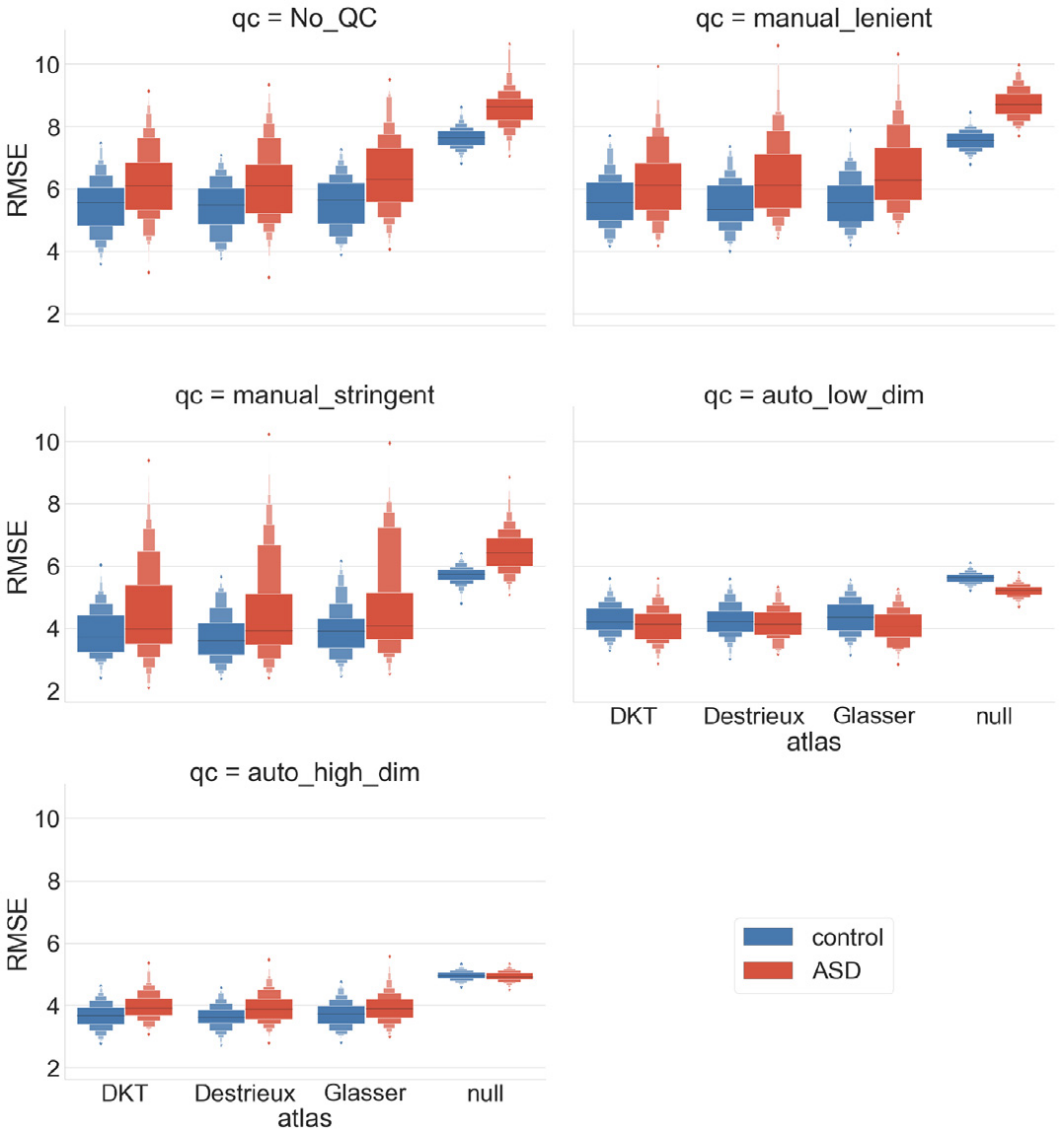
Task-driven individual (TD-I) analysis. Individual age prediction with various parcellations and QC levels stratified by diagnosis. Performance (measured by Root Mean Square Error, RMSE) is cross-validated using a random forest model over 100 shuffle-split iterations.

Parcellation-based comparisons show similar RMSE performance despite the differences in granularity of regions and the consequent number of input features to the ML models (see Fig. 9). The RMSE trends with respect to QC are also consistent, with both stringent and automatic QC reducing the mean RMSE and the latter yielding a much tighter distribution of error. The null model shows lower expected error for the control cohort compared to the ASD, except for the analyses based on automatic QC, where this expectation is reversed.

### ROI importance from random forest

**Figure 10:**
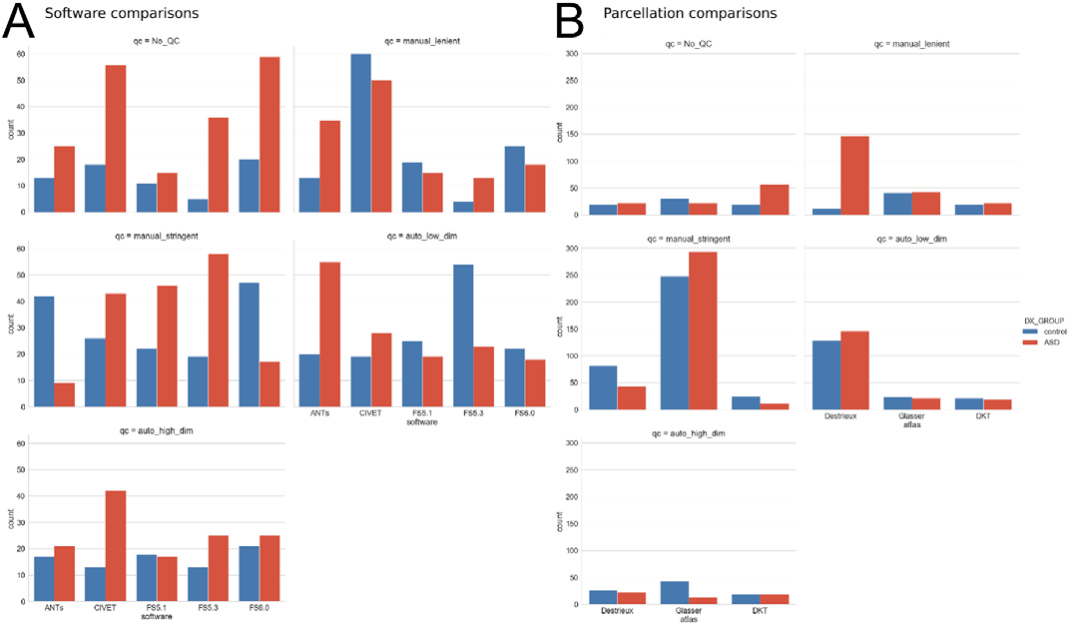
Predictive feature set count with various (A) software and (B) parcellations for different QC levels stratified by diagnosis. Optimal predictive features are selected using cross-validated recursive feature elimination procedure.

The cross-validated recursive feature elimination (RFE) procedure yields drastically different feature sets across software tools (see Fig. 10). Overall all software tools require a small number of features for age prediction of control participants (*n*ϵ[3, 20]) compared to ASD participants (*n*ϵ[41, 60]). RFE seems to be very sensitive to the QC procedures as these yield different feature sets with no apparent consistent trends for controls or ASD cohorts. The parcellation comparisons also show varied selection of features. Despite the larger number of regions for Destrieux and Glasser parcellations, the number of predictive features remains relatively small. The sensitivity to QC procedure appears to be reflected in the parcellation analysis as evidenced by large spikes in feature counts for both control and ASD cohorts.

### Validation analysis

For the HCP dataset, the feature comparisons based on DKT parcellation yielded a mean Pearson correlation of 0.66 between CIVET2.1 and FS6.0 (ABIDE: *r* = 0.52). The regions exhibiting low correlations were also consistent with ABIDE analysis, and comprised cingulate regions, orbitofrontal regions, EC, pericalcarine, and insula.

## Discussion

In this work, we aimed to assess the reproducibility of phenotypic features and subsequent findings subjected to preprocessing pipeline variation along 3 axes: (i) image-processing tool, (ii) anatomical priors, and (iii) QC. We emphasize that the goal here is not to deliberate specific biological and individual interpretation from the analyses but rather to highlight the differences among the findings themselves, a key piece of information for the large community of researchers using anatomical brain imaging in their studies.

In the TF-N analysis, we observe a weak ROI-wise correlation across software pairs (see Fig. 3). Although software-specific biases are expected in biological phenotypic estimates, the level of diminished correlation is striking. One can explain this performance for the comparisons involving ANTS because it is the only software that operates in the voxel (volume) space. However, a similarly poor performance is seen with CIVET and FS, both of which operate in a vertex (surface) space for cortical thickness estimation. Because individual ROI-based measures are frequently used in the downstream mass-univariate models, the lack of consensus across software tools is likely to yield different results. Moreover, the varying ROI covariance patterns across the software tools (see Fig. 4) suggest weak multivariate similarity, which again strongly increases the dependence of findings and biological interpretations on the software choice. This variability can be potentially explained by the differences in underlying biological assumptions that dictate several software-specific metrics. For instance, there are several ways to estimate cortical thickness as distance between GM and WM surfaces. It appears that the algorithmic specificities of CIVET give rise to more symmetric patterns within cortical ROIs as seen in Fig. 4. Last, the lack of impact from QC suggests that these effects are systemic and not driven by outliers.

In the TF-I analysis, software tool–specific t-SNE similarity is encouraging and expected. The t-SNE embeddings also highlight stronger differences between software tools compared to the differences in diagnostic groups (see Fig. 5). This partly explains the high difficulty in training generalizable ML models across studies using different preprocessing pipelines. The poor CC with the commonly used Ward linkage criterion is alarming (see Table 4). Given that data-driven clustering is a typical practice to identify subgroups of patients or define meaningful biomarkers [49, 50], clustering membership that is highly sensitive to the preprocessing pipeline may go undetected by the stability tests performed on the final set of processed features.

In the TD-N analysis, the software and parcellation comparisons show relatively consistent spatial associations for the age regression models (see Figs 7 and 8). There are some software-specific regional peculiarities: e.g., cortical thickness of entorhinal regions seems to have significant association with age for ANTS and FS5.1 but not in other software. Then, CIVET uniquely shows lack of association at insular regions. ANT and CIVET also show much higher sensitivity to QC procedures. These sensitivities should be noted because they could suggest methodological limitations or bias in the software. The overall cortex-wide association of thickness with age is expected because various studies have reported the same in healthy and ASD populations [44, 46, 51–53]. Direct comparison with other studies is challenging owing to differences in the underlying statistical models, which produce varying topologies of widespread associations, and the direction of change in the cortical thickness. The results in this work suggest that the lack of strong ROI (univariate) correlation between a pair of software tools does not affect the task-driven mass-univariate analysis. However, we note that this is highly specific to the task at hand, as well as model selection procedures, which are beyond the scope of this work. We speculate that localized effects are likely to be more sensitive to the univariate pairwise relationships, and therefore a novel biological finding must be reported with high scrutiny to exclude pipeline specificities. Towards this cause, reporting findings with multiple parcellations defined with different underlying assumptions (biological: DKT, Destrieux vs data-driven: Glasser) offering a range of spatial granularities can help to reaffirm the regional effects.

In the TD-I analysis, age prediction with RF is stable subject to software and parcellation variations (see Figs 8 and 9). The RMSE performance of 3.8–4.7 years is comparable to the similar previous age prediction studies [17, 43, 44], which report RMSE in ranges of 6–12 years or mean absolute error of 1.7–1.95 years. The stability of performance could potentially be attributed to the relatively large sample sizes. It is encouraging to see that biological noise does not induce large variations into individual predictions. It is also important to note the impact of QC on the model performance and the null distributions for a given population (i.e., controls vs ASD). These alterations in the expected null performance need to be reported in order to fairly evaluate the improvements offered by a novel model on a given sample. Although RF seems to be stable for individual predictions, the feature importance assessments by the same model are highly variable (see Fig. 10). One explanation for this behaviour could be that in the presence of noisy biological features, ML models assign a relatively flat distribution of importance to the features. Variation in feature sets or sample sizes, as dictated by the selected preprocessing pipeline, would thus yield a drastically different feature ranking in a given iteration of the analysis. This needs to be taken into account if ML models are used to make biological inferences.

The validation analysis with HCP allowed us to replicate our feature correlation findings on an independent dataset. Similar to the ABIDE analysis, HCP data showed consistent low correlation between the ROI thickness values produced by FS6.0 and CIVET2.1. Moreover, there is a large overlap in the regions (i.e., cingulate regions, orbitofrontal regions, EC, and insula) exhibiting the low correlations. This suggests that the low correlations are mainly driven by the algorithmic differences and not by the dataset. The pericalcarine was the exception to this common regional subset, having a low correlation only in the HCP dataset, possibly due to dataset-specific peculiarities. Nevertheless this highlights the need for larger meta-analyses to identify tool-specific and dataset-specific variability in findings.

### Limitations

Although in this work we aimed at assessing the impact of pipeline vibration along 3 different axes, we only considered a subset of permutations in the analysis. This was primarily due to practical reasons such as the lack of availability of common parcellation definitions for all software tools. Therefore we could not compare software tools with Destrieux and Glasser parcellations. We also note that we did not disambiguate effect measurement noise, typically estimated with a test-retest subsample in a dataset. This is because the previous studies have shown high reliability of cortical thickness measures and subsequent derived features [54, 55]. We also limited the scope of this work to structural features and did not consider functional or diffusion measures. With the increasing popularity of sophisticated, derived measures from highly flexible functional preprocessing pipelines with a multitude of design parameters, it is critical to understand and quantify the inherent variability and its impact on downstream findings. We defer this endeavour to future studies and refer to Bowring et al. [6] for some progress in this direction.

## Conclusions

This work highlights the variability introduced by preprocessing pipelines, which is only a part of the larger issue of reproducibility in computational neuroimaging. We understand that the computational burden of comparative analyses such as described here can be infeasible in many studies. This necessitates undertaking of large meta-analytic studies to understand software-specific biases for various populations stratified by demographic characteristics and diseases. At the single-study level, we encourage the community to process data with different tools as much as possible and report variation of the results. We also propose to systematically report positive and negative results with different parcellations. This will improve the level of confidence in the findings and help to better elucidate the spatial granularity associated with the effect of interest, while facilitating comparisons of common atlases across tools. Last, we also recommend assessing the sensitivity of findings against varying degrees of stringency for the QC criteria. Only with widespread adoption of rigorous scientific methodology and accessible informatics resources to replicate and compare processing pipelines can we address the compounding problem of reproducibility in the age of large-scale, data-driven computational neuroscience. The availability of containerized and well-documented pipelines, together with the necessary computing resources, will mitigate the variability of results observed and direct the community towards understanding these differences, as well as further develop methodological validation and benchmarking.

## Data Availability

All supporting material, including csv data and code to generate the figures, can be found at [23]. See also sections Participants, CIVET, ANTS, and Freesurfer for the exact version and location of these publicly available datasets and software. Snapshots of our code and other supporting data can be openly found in the *GigaScience* respository, GigaDB [56].

## Additional Files

**Supplementary Figure S1:** TF-N analysis for the HCP dataset. Correlation between cortical thickness values for CIVET2.1 and FS6.0 measured independently over ROIs for control and ASD groups. The vertical lines represent the mean correlation across all ROIs, defined using DKT parcellation.

**Supplementary Figure S2:** . TF-I analysis for the HCP dataset. t-SNE plot showing difference between individual embeddings for CIVET2.1 and FS6.0 software.

## Competing Interests

The authors declare that they have no competing interests.

## Abbreviations

ABIDE: Autism Brain Imaging Data Exchange; ANTS: Advanced Normalization ToolS; ASD: autism spectrum disorder; CC: clustering consistency; csv: comma-separated values; DKT: Desikan-Killiany-Tourville; EC: entorhinal cortex; FS: Freesurfer; GLM: general linear model; GM: grey matter; HCP: Human Connectome Project; ML: machine learning; MRI: magnetic resonance imaging; NITRC: Neuroimaging Tools and Resources Collaboratory; PeEC: perirhinal ectorhinal cortex; PIR: piriform cortex; QC: quality control; RF: random forest; RFE: recursive feature elimination; RMMMD: random min-max multiple deletion; RMSE: root mean square error; ROI: region of interest; STG: superior temporal gyrus; TF: task free (-I: individual, -N: neurobiological); TD: task driven (-I: individual, -N: neurobiological); t-SNE: t-distributed stochastic neighbour embedding; WM: white matter.

## Funding

This work was partially funded by the National Institutes of Health (NIH) NIH-NIBIB P41 EB019936 (ReproNim), NIH-NIMH R01 MH083320 (CANDIShare), and NIH RF1 MH120021 (NIDM), the National Institute of Mental Health of the NIH under Award No. R01MH096906 (Neurosynth), as well as the Canada First Research Excellence Fund, awarded to McGill University for the Healthy Brains for Healthy Lives initiative and the Brain Canada Foundation with support from Health Canada.

## Authors' Contributions

N.B conceived the study, implemented the analyses, wrote the manuscript.

K.H. A.B., E.D. participated in the data analyses and revision of the manuscript.

E.W.D, S.T.B, G.A.D, E.D. A.D., M.C, C.M.T.G, B.M, D.N.K participated in the interpretation of the results and the revision of the manuscript.

J.-B.P. participated in the conception of the study, analyses interpretation and manuscript writting.

## Supplementary Material

giaa155_GIGA-D-20-00232_Original_Submission

giaa155_GIGA-D-20-00232_Revision_1

giaa155_GIGA-D-20-00232_Revision_2

giaa155_Response_to_Reviewer_Comments_Original_Submission

giaa155_Response_to_Reviewer_Comments_Revision_1

giaa155_Reviewer_1_Report_Original_Submission Ariel Rokem, PhD -- 8/28/2020 Reviewed

giaa155_Reviewer_1_Report_Revision_1 Ariel Rokem, PhD -- 11/4/2020 Reviewed

giaa155_Reviewer_2_Report_Original_Submission Yu-Feng Zang -- 9/4/2020 Reviewed

giaa155_Supplemental_File
