# Supplementary material for: Understanding the impact of preprocessing pipelines on neuroimaging cortical surface analyses
Source: Gigascience. 2021 Jan 22;10(1):giaa155. doi: 10.1093/gigascience/giaa155 (PMC7821710; doi:10.1093/gigascience/giaa155)
Supplement: giaa155_GIGA-D-20-00232_Original_Submission [file giaa155_giga-d-20-00232_original_submission.pdf]

GigaScience

Understanding the impact of preprocessing pipelines on neuroimaging cortical surface analyses

--Manuscript Draft--

**Manuscript Number:**	GIGA-D-20-00232
**Full Title:**	Understanding the impact of preprocessing pipelines on neuroimaging cortical surface analyses
**Article Type:**	Research
**Funding Information:**	National Institutes for Water Resources (US) (EB019936) — Dr. Jean-Baptiste Poline<br>Healthy Foods, Healthy Lives Institute, University of Minnesota (US) — Dr. Jean-Baptiste Poline<br>NIMH (R01MH096906) — Dr. Jean-Baptiste Poline<br>National instute of Health (MH120021) — Dr. Jean-Baptiste Poline

Abstract:

The choice of preprocessing pipeline introduces variability in neuroimaging analyses that affects the reproducibility of scientific findings. Features derived from structural and functional MR imaging data are sensitive to the algorithmic or parametric differences of preprocessing tasks, such as image normalization, registration, and segmentation to name a few. Therefore it is critical to understand and potentially mitigate the cumulative biases of pipelines in order to distinguish biological effects from methodological variance. Here we use an open structural MR imaging dataset (ABIDE), supplemented with the Human Connectome Project (HCP), to highlight the impact of pipeline selection on cortical thickness measures. Specifically, we investigate the effect of 1) software tool (e.g. ANTs, CIVET, FreeSurfer), 2) cortical parcellation (DKT, Destrieux, Glasser), and 3) quality control procedure (manual, automatic). We divide our statistical analyses by 1) method type, i.e. task-free (unsupervised) versus task-driven (supervised), and 2) inference objective, i.e. neurobiological group differences versus individual prediction. Results show that software, parcellation, and quality control significantly impact task-driven neurobiological inference. Additionally, software selection strongly impacts neurobiological and

	individual task-free analyses, and quality control alters the performance for the individual-centric prediction tasks. This comparative performance evaluation partially explains the source of inconsistencies in neuroimaging findings. Furthermore, it underscores the need for more rigorous scientific workflows and accessible informatics resources to replicate and compare preprocessing pipelines to address the compounding problem of reproducibility in the age of large-scale, data-driven computational neuroscience.
**Corresponding Author:**	Jean-Baptiste Poline<br>McGill University<br>Montreal, Quebec CANADA
**Corresponding Author Secondary Information:**	
**Corresponding Author's Institution:**	McGill University
**Corresponding Author's Secondary Institution:**	
**First Author:**	Nikhil Bhagwat
**First Author Secondary Information:**	
**Order of Authors:**	Nikhil Bhagwat
	Amadou Barry
	Erin W. Dickie
	Shawn Brown
	Gabriel Devenyi
	Koji Hatano
	Elizabeth DuPre
	Alain Dagher
	Mallar Chakravarty
	Celia Greenwood
	Bratislav Misic
	David Kennedy
	Jean-Baptiste Poline
**Order of Authors Secondary Information:**	
**Additional Information:**	
**Question**	**Response**
Are you submitting this manuscript to a special series or article collection?	No
**Experimental design and statistics**	Yes

Full details of the experimental design and statistical methods used should be given in the Methods section, as detailed in our Minimum Standards Reporting Checklist. Information essential to interpreting the data presented should be made available in the figure legends.<br><br>Have you included all the information requested in your manuscript?	
**Resources**<br><br>A description of all resources used, including antibodies, cell lines, animals and software tools, with enough information to allow them to be uniquely identified, should be included in the Methods section. Authors are strongly encouraged to cite Research Resource Identifiers (RRIDs) for antibodies, model organisms and tools, where possible.<br><br>Have you included the information requested as detailed in our Minimum Standards Reporting Checklist?	Yes
**Availability of data and materials**<br><br>All datasets and code on which the conclusions of the paper rely must be either included in your submission or deposited in publicly available repositories (where available and ethically appropriate), referencing such data using a unique identifier in the references and in the "Availability of Data and Materials" section of your manuscript.<br><br>Have you have met the above requirement as detailed in our Minimum Standards Reporting Checklist?	Yes

Manuscript Click here to access/download;Manuscript;Bahwat-et-al-2020.pdf

Understanding the impact of preprocessing pipelines on neuroimaging cortical surface analyses

Nikhil Bhagwat[1,✉], Amadou Barry[2], Erin W. Dickie[3], Shawn T. Brown[1], Gabriel A. Devenyi[4,5], Koji Hatano[1], Elizabeth DuPre[1], Alain Dagher[1], M. Mallar Chakravarty[4,5,10], Celia M. T. Greenwood[2,8,9], Bratislav Misic[1], David N. Kennedy[7], and Jean-Baptiste Poline[1,6,8,✉]

[1]Montreal Neurological Institute Hospital, McGill University, Montreal, QC, Canada
[2]Lady Davis Institute for Medical Research, McGill University, Montreal, QC, Canada
[3]Kimel Family Translational Imaging-Genetics Research Lab, CAMH, Toronto, ON, Canada
[4]Computational Brain Anatomy Laboratory, Douglas Mental Health Institute, Verdun, QC, Canada
[5]Department of Psychiatry, McGill University, Montreal, QC, Canada
[6]Department of Neurology and Neurosurgery, McGill University, Montreal, QC, Canada
[7]Child and Adolescent Neurodevelopment Initiative, University of Massachusetts, Worcester, USA
[8]Ludmer Centre for Neuroinformatics Mental Health, McGill University, Montreal, QC, Canada
[9]Gerald Bronfman Department of Oncology; Department of Epidemiology, Biostatistics Occupational Health; Department of Human Genetics, McGill University, Montreal, QC, Canada
[10]Department of Biomedical Engineering, McGill University

The choice of preprocessing pipeline introduces variability in neuroimaging analyses that affects the reproducibility of scientific findings. Features derived from structural and functional MR imaging data are sensitive to the algorithmic or parametric differences of preprocessing tasks, such as image normalization, registration, and segmentation to name a few. Therefore it is critical to understand and potentially mitigate the cumulative biases of pipelines in order to distinguish biological effects from methodological variance. Here we use an open structural MR imaging dataset (ABIDE), supplemented with the Human Connectome Project (HCP), to highlight the impact of pipeline selection on cortical thickness measures. Specifically, we investigate the effect of 1) software tool (e.g. ANTs, CIVET, FreeSurfer), 2) cortical parcellation (DKT, Destrieux, Glasser), and 3) quality control procedure (manual, automatic). We divide our statistical analyses by 1) method type, i.e. task-free (unsupervised) versus task-driven (supervised), and 2) inference objective, i.e. neurobiological group differences versus individual prediction. Results show that software, parcellation, and quality control significantly impact task-driven neurobiological inference. Additionally, software selection strongly impacts neurobiological and individual task-free analyses, and quality control alters the performance for the individual-centric prediction tasks. This comparative performance evaluation partially explains the source of inconsistencies in neuroimaging findings. Furthermore, it underscores the need for more rigorous scientific workflows and accessible informatics resources to replicate and compare preprocessing pipelines to address the compounding problem of reproducibility in the age of large-scale, data-driven computational neuroscience.

Keywords: neuroimaging, reproducibility, cortical thickness, preprocessing pipelines

Correspondence: *nikhil153@gmail.com, jean-baptiste.poline@mcgill.ca*

Introduction

Reproducibility, a presumed requisite of any scientific experiment, has recently been under scrutiny in the field of computational neuroscience [1–7]. Specifically, replicability and generalizability of several neuroimaging pipelines and the subsequent statistical analyses have been questioned, potentially due to insufficient sample size [8], imprecise or flexible methodological and statistical apriori assumptions [9–11], and poor data/code sharing practices [12,13]. Broadly speaking, reproducibility can be divided in two computational goals [14]. The first goal is replicability, which implies that a re-executed analysis on the identical data should always yield the same results. The second goal pertains to generalizability, which is assessed by comparing the scientific findings under variations of data and analytic methods. Typically, findings are deemed generalizable when similar (yet independent) data and analysis consistently support the experimental hypothesis. This in turn raises the issue of defining what constitutes "similar" data and analytic methodology. Nonetheless, traditionally experimental validation on independent datasets has been utilized to assess generalizability. However, as the use of complex computational pipelines has become an integral part of modern neuroimaging analysis [15], comparative assessment of these pipelines and their impact on the generalizability of findings deserves more attention.

We present a comparative assessment of multiple structural neuroimaging preprocessing pipelines on the Autism Brain Imaging Data Exchange (ABIDE), a publicly accessible dataset comprising healthy controls and individuals with autism spectrum disorder (ASD) [18]. A few studies have previously highlighted the variability in neuroimaging analyses introduced by the choice of a preprocessing pipeline for structural MR images [16,17], however they have not focused on the relative impact of analysis tools, quality control, and parcellations on the consistency of results. The inconsistencies in the results arise from several algorithmic and parametric differences that exist in the preprocessing tasks, such as image normalization, registration, segmentation, etc. within pipelines. It is critical to understand and potentially mitigate

the cumulative biases of the pipelines to disambiguate biological effect from methodological variance. We further replicate our findings on the Human Connectome Project (HCP) data.

For this purpose, we propose a comprehensive investigation of the impact of pipeline selection on cortical thickness measures, a widely used (3129 hits on PubMed and 42,200 hits on Google Scholar for "cortical thickness" AND "Magnetic resonance imaging" search query), fundamental phenotype, and its statistical association with biological age. We limit the scope of pipeline variation to three axes of parameter selection: 1) image processing tool, 2) anatomical priors, 3) quality control (see Fig 1). The impact of the variation is measured on two types of statistical analyses, namely: 1) neurobiological inference carried out using general linear modeling (GLM) techniques at the group level; and 2) individual predictions from machine-learning (ML) models. We note that here the focus is on the preprocessing stages of a computational pipeline, and the impact of dataset and statistical model selection is thus out of the current scope. Our goal is not to explain potential differences in results or establish criteria to rank pipelines or tools, but to document the pipeline effect and provide best practice recommendations to the neuroscience community with respect to pipeline variation, also referred to as pipeline vibration effects.

Although here we do not focus on identifying biological differences between ASD case and control groups, we use the case-control samples to gain insight into the effect of diagnosis on reproducibility analysis - which is a critical evaluation for clinical applications. Additionally, we use a data sample from the HCP as a validation dataset (Van Essen DC et al. 2013) to assess if our findings replicate on an independent dataset. Note that the scope of this secondary analysis is limited to a proof of concept dataset comparison.

We organize our comparative assessments on the ABIDE dataset as follows. We report comparisons across the three aforementioned axes of variation. This comprises five neuroimaging preprocessing tools: 1) FreeSurfer 5.1, 2) FreeSurfer 5.3, 3) FreeSurfer 6.0, 4) CIVET 2.1.0, and 5) ANTs; three anatomical priors (i.e. cortical parcellations): 1) Desikan-Killiany-Tourville, 2) Destrieux, and 3) Glasser; and five quality control (QC) procedures 1) No QC 2) manual lenient 3) manual stringent, 4) low-dimensional automatic outlier detection (i.e. <500 ROIs), and 5) high-dimensional automatic outlier detection (i.e. > 100k vertices). The entire combinatorial set of comparisons (5 software x 3 parcellations x 5 QC) is not feasible due to practical limitations (described later), and therefore we report results for five tools procedures and three atlases across five quality control procedures (5 software + 3 parcellations) x 5 QC, as shown by the connecting arrows in Fig 1. We use these 40 preprocessed data with four types of statistical analyses based on a method type (i.e. task-free vs. task-driven) and an inference objective (neurobiological vs. individual), as described in detail in the methods.

Materials and Methods

Participants. Participants from the ABIDE dataset were used for this study [18]. The ABIDE 1 dataset comprises 573 control and 539 autism spectrum disorder (ASD) individuals from 16 international sites. The neuroimaging data of these individuals were obtained from the ABIDE preprocessing project [19], the Neuroimaging Tools and Resources Collaboratory (NITRC) (http://fcon_1000.projects.nitrc.org/indi/abide/abide_I.html), and the DataLad repository (http://datasets.datalad.org/?dir=/abide/RawDataBIDS). Different subsets of individuals were used for various analyses based on 1) specific image processing failures, 2) need for a common sample set for software tool comparison, and 3) quality control procedures. The demographic description of these subsets is provided in Table 1, and Figure 2. The complete lists of subjects can be obtained from the code repo: https://github.com/neurodatascience/compare-surf-tools

MR Image processing and cortical thickness measurements.

FreeSurfer. FreeSurfer (FS) delineates the cortical surface from a given MR scan and quantifies thickness measurements on this surface for each brain hemisphere [20,21]. The default pipeline consists of 1) affine registration to the MNI305 space [22]; 2) bias field correction; 3) removal of skull, cerebellum, and brainstem regions from the MR image; 3) estimation of white matter surface based on MR image intensity gradients between the white and grey matter; and 4) estimation of pial surface based on intensity gradients between the grey matter and cerebrospinal fluid (CSF). The distance between the white and pial surfaces provides the thickness estimate at a given location of cortex. For detailed description refer to [23]. The individual cortical surfaces are then projected onto a common space (i.e. fsaverage) characterized by 163,842 vertices per hemisphere to establish inter-individual correspondence.
In this work, the cortical thickness for each MR image was computed using FS 5.1, 5.3, and 6.0 versions. The FS5.1 measurements were obtained from the ABIDE preprocessing project [19]. Standard recon-all pipeline with "-qcache" flag was used to process and resample the images onto common (fsaverage) space. The FS5.3 measurements were extracted using the standard ENIGMA cortical thickness pipeline [24]. Lastly, the FS6.0 measurements were obtained using the standard recon-all pipeline with "-qcache" flag as well. Compute Canada [25] and CBRAIN [26] computing infrastructures were used for processing of FS5.3 and FS6.0 data.

CIVET. CIVET 2.1 (http://www.bic.mni.mcgill.ca/ServicesSoftware/CIVET-2-1-0-Introduction) preprocessing was performed on the data obtained from NITRC. The standard CIVET pipeline consists of 1) N3 bias correction [27]; 2) affine registration to the MNI ICBM 152 stereotaxic space; 3) tissue classification into white matter (WM), grey matter

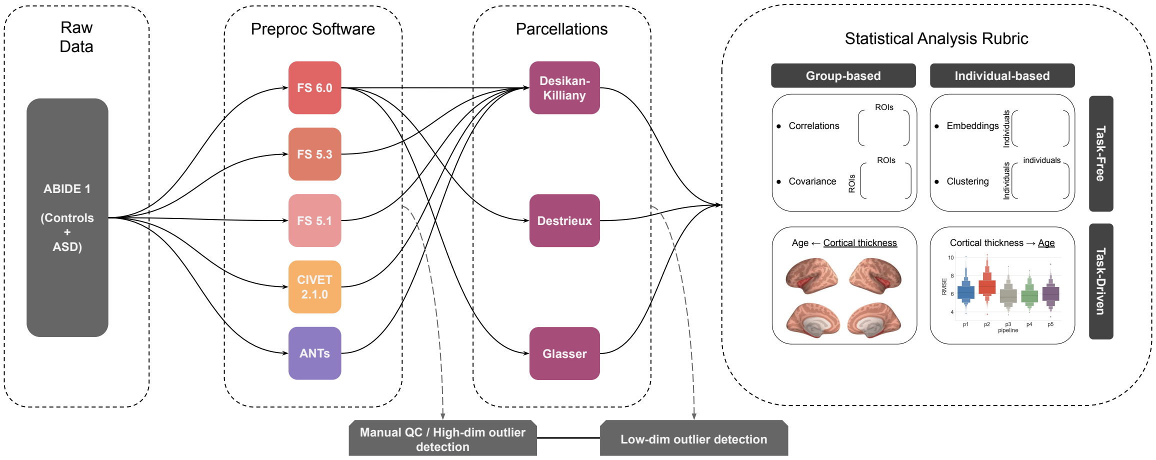

Fig. 1. Preprocessing pipeline building blocks and potential permutations for a typical structural MR image analysis. Only a subset of the possible pipelines is analyzed and shown with arrows. Note that manual quality control and automatic outlier detection can be performed at various stages.

Comparisons	QC	Diagnosis	Subjects (N)	Age (mean, sd)	Sex (M/F)
Software tools	No QC(N=778)	Controls	415	17.8, 7.7	346/69
		ASD	363	18.3, 8.7	320/43
	Lenient Manual(N=748)	Controls	407	17.8, 7.6	338/69
		ASD	341	18.4, 8.8	300/41
	Stringent Manual(N=194)	Control	113	15.6, 5.5	93/20
		ASD	81	16.2, 5.8	71/10
	Auto QC low-dim(N=683)	Controls	371	16.2, 5.4	309/62
		ASD	312	15.9, 5.0	276/36
	Auto QC high-dim(N=662)	Controls	356	15.6, 5.0	293/63
		ASD	306	15.7, 4.9	269/37
Parcellations	No QC(N=1047)	Controls	552	17.0, 7.5	456/96
		ASD	495	17.1, 8.4	436/59
	Lenient Manual(N=975)	Controls	525	17.1, 7.5	430/95
		ASD	450	17.4, 8.6	395/55
	Stringent Manual(N=240)	Controls	137	15.0, 5.6	112/25
		ASD	103	16.1, 6.3	91/12
	Auto QC low-dim(N=961)	Controls	516	15.6, 5.6	422/94
		ASD	445	15.0, 5.1	390/55
	Auto QC high-dim(N=912)	Controls	483	15.0, 4.9	393/90
		ASD	429	14.9, 4.9	377/52

Table 1. Subject demographics for different analyses

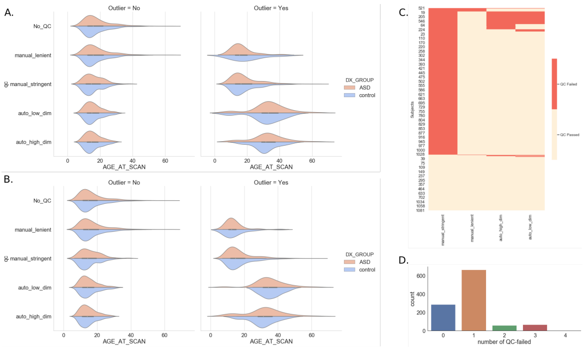

Fig. 2. Age distributions for sample subsets used for (A) software comparison and (B) parcellation comparison analyses. See Table 1 for sample sizes. Failed QC overlap across manual QC and automatic outlier detection procedures is show in (C). Distribution of total outlier count (sum) based on four possible manual QC and automatic outlier detection procedures is shown in (D)

(GM) and cerebrospinal fluid; 4) brain splitting into left and right hemispheres for independent surface extraction; 5) estimation of WM, pial, and GM surfaces. The cortical thickness is then computed using the distance (i.e. Tlink metric) between WM and GM surfaces at 40,962 vertices per hemisphere.

ANTs. The MR imaging dataset preprocessed with ANTs ("RRID:SCR_004757, version May-2017") was obtained from the ABIDE preprocessing project [19]. The detailed description of ANTs cortical thickness pipeline can be found here [16]. Briefly, the ANTs pipeline consists of 1) N4 bias correction [28]; 2) brain extraction; 3) prior-based segmentation and tissue-based bias correction; and 4) Diffeomorphic registration-based cortical thickness estimation [29].

Software Tool		
Analysis type	Neurobiology (N)	Individual (I)
Task free (TF)	Feature correlations and covariance	Individual embeddings and clustering
Task driven (TD)	ROI ∼Age + covars	Age ← ROIs + covars
Cortical Parcellation		
Analysis type	Neurobiology (N)	Individual (I)
Task free (TF)	N/A	N/A
Task driven (TD)	ROI ∼Age + covars	Age ← ROIs + covars
Quality Control		
Analysis type	Neurobiology (N)	Individual (I)
Task free (TF)	N/A	N/A
Task driven (TD)	ROI ∼Age + covars	Age ← ROIs + covars

Table 2. 2x2 rubric showing types of analysis performed for each axis of variation

One key differentiating aspect of ANTs is that it employs quantification of cortical thickness in the voxel-space, unlike FreeSurfer or CIVET, which operate with vertex-meshes.

Cortical parcellations. The regions of interest (ROI) were derived using three commonly used cortical parcellations, namely 1) Desikan-Killiany-Tourville (DKT) [30], 2) Destrieux [31], and 3) Glasser [32]. DKT parcellation consists of 31 ROIs per hemisphere and is a modification of the Desikan–Killiany protocol [33]) to improve cortical labeling consistency. DKT label definitions are included in all three FreeSurfer (FS), CIVET, and ANTs pipelines, which allows the comparison of cortical phenotypic measures across these tools. The Destrieux parcellation is a more detailed anatomical parcellation proposed for a precise definition of cortical gyri and sulci. The Destrieux parcellation comprises 74 ROIs per hemisphere, and is also available in the FS pipeline. In contrast to these structural approaches, the Glasser parcellation was created using multimodal MR acquisitions from 210 HCP subjects [34] with 180 ROIs per hemisphere. Glasser label definitions are available in the "fsaverage" space (https://doi.org/10.6084/m9.figshare.3498446.v2), i.e. the common reference space used by FreeSurfer, allowing comparisons across multiple parcellations.

Quality Control. We employed manual (i.e. visual) and automatic (statistical outlier detection) procedures to investigate the effect of quality control (QC) on thickness distributions derived from combinations of the different software tools and cortical parcellations. The manual quality checks were performed on the extracted cortical surfaces by two independent expert raters [35,36]. The two raters used different criteria for assessing the quality of surface delineation. This in turn yielded two lists of QC-passed subjects from "lenient" and "stringent" criteria. We note that these lenient and stringent QC lists were generated independently using FS and CIVET images, respectively; and then applied to all pipeline variations. The automatic quality control was performed using an outlier detection algorithm based on a random min-max multiple deletion (RMMMD) procedure (Barry et al. in preparation). The RMMMD algorithm is a high-dimensional extension of Cook's influence measure to identify influential observations. The outlier detection method was applied separately to high-dimensional vertex-wise output and low-dimensional aggregate output based on cortical parcellations for each software and parcellation choice.

Statistical Analysis . We categorize the downstream statistical analyses into a 2x2 design. The first factor consists of either 1) unsupervised, task-free (TF) analyses or 2) supervised, task-driven (TD) analyses. The second factor corresponds to either 1) neurobiological (N) tasks investigating the biological effect across groups of individuals or 2) individual (I) tasks predicting individual-specific states (see Table 2). The task-free, neurobiologically oriented analyses (TF-N) aim at quantifying similarity of preprocessed features (i.e. ROI-wise cortical thickness values) without the explicit constraint of an objective function. Task-driven, neurobiologically oriented analyses (TD-N) quantify feature similarity in the context of a general linear model (GLM) framework. Individually oriented analyses formulate the duality of neurobiological analyses, with a focus on individual similarity in task-free (TF-I) and task-driven (TD-I) contexts.

Previous work has reported varying degrees of association and predictability of age from cortical thickness measures in neurotypical and ASD cohorts [37–41]. We therefore selected biological age as our objective for the task-driven (TD) analyses. Although other clinical variables (e.g. diagnosis) could be used, availability and unambiguity of age quantification across datasets simplifies comparison of the different analyses.

For TF-N analysis we evaluate the pairwise correlation and covariance of features using Pearson's r metric. For TF-I analysis, we assess individual similarity using t-SNE and hierarchical clustering with Euclidean distance and Ward's linkage metrics. For TD-N analysis, we build a GLM to associate cortical thickness and biological age with sex and data collection site as covariates. For TD-I analysis, we train a

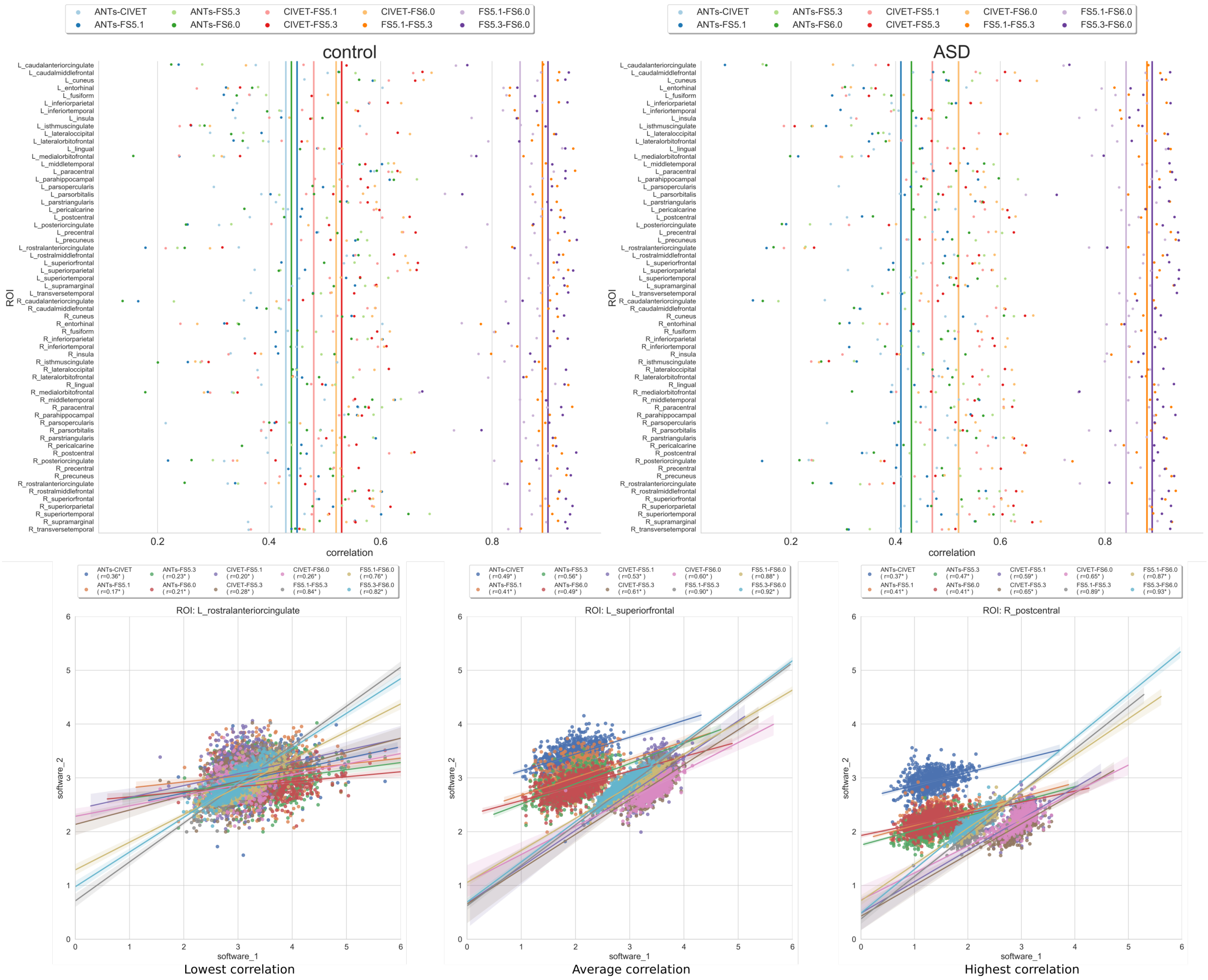

Fig. 3. Task Free - Neurobiology (TF-N) analysis: Top) Correlation between cortical thickness values for software pairs measured independently over ROIs for control and ASD groups. The vertical lines represent the mean correlation across all ROIs. The ROIs are defined using Desikan-Killiany-Tourville (DKT) parcellation. Bottom) Distribution of cortical thickness values for exemplar ROIs with lowest, average, and highest median correlation across software pairs.

random forest (RF) model for age prediction using cortical thickness, sex, and data collection site as predictors. Of note, we also assess the importance assigned to cortical features by the RF model. Machine learning (ML) model performance and feature importance is assessed within 100 iterations of a shuffle-split cross-validation paradigm.

We also note that not all pipeline variations can be assessed easily within this to 2x2 statistical analyses design. As mentioned before we only analyze a subset ((5+3)x5) of possible pipeline variations, and compare the five software tools using common DKT parcellation. Tool comparison with Destrieux and Glasser parcellations is not trivial due to their unavailability for CIVET and ANTs. This also limits our comparison across three parcellations solely with FreeSurfer 6.0. We do however compare all five QC procedures with these combinations. The analyses performed in this work are provided in Table 2. The code used for the analyses is available here: https://github.com/neurodatascience/compare-surf-tools.

Validation Study. The T1w images of 1108 individuals from the HCP dataset [42] were successfully preprocessed using FS 6.0 and CIVET 2.1 respectively, and average cortical thickness measurements in the DKT ROIs were obtained. Identical to the ABIDE analysis, we evaluated the pairwise correlations and covariance of features between CIVET 2.1 and FS 6.0 using Pearson's r metric, then we compared it using the same approach as for the ABIDE dataset.

Results

Task-free neurobiological (TF-N) analysis. Feature comparisons across the five software tools are performed using common DKT parcellation. The pairwise comparisons between software tools are performed based on the ROI-wise Pearson correlations between thickness measures produced by each tool (See Figure 3, Table 3). The pairwise comparisons between FS, CIVET, and ANTs tools show very little similarity with correlation values averaged over all regions remaining low ($r \epsilon [0.39, 0.52]$). The comparisons between different versions of FS show relatively better average correlation performance ($r \epsilon [0.83, 0.89]$). Stratifying comparisons by diagnosis does not improve correlation. ROI specific performance shows the lowest median correlation for the left rostral-anterior-cingulate (r=0.27), left and right isthmus-cingulate (r=0.29,0.31) regions, and the highest median correlation for the left cuneus (r=0.63), right postcen-

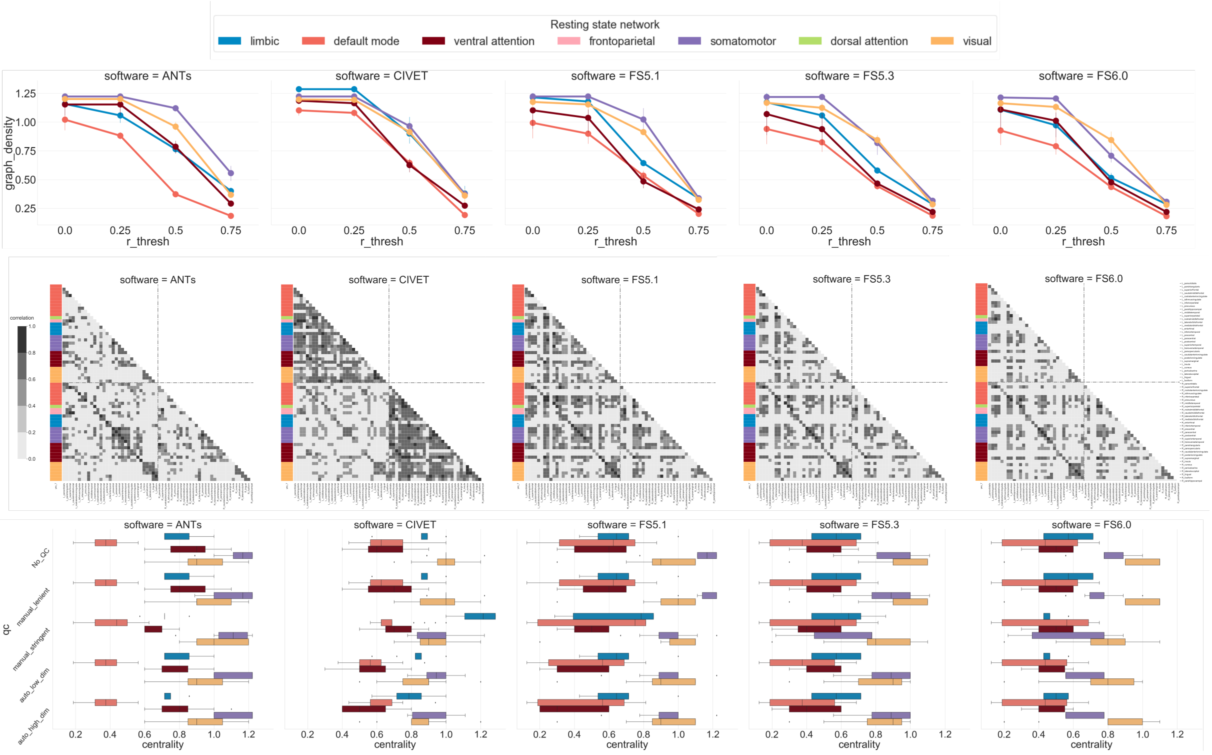

Fig. 4. Task-Free neurobiological (TF-N) analysis: Top) Graph density for different correlation cutoff thresholds used for constructing a structural network. The error bars show variation due to the QC procedure. Middle) Structural covariance of each software measured as inter-ROI correlation with cutoff value of 0.5. For simplicity, the covariance plot is generated with original data. The covariance patterns are grouped by Yeo resting state networks membership. Bottom) Distribution of regional degree-centrality metric per Yeo network for each software with different QC procedures. Note that fronto-parietal and dorsal attentional networks are excluded from some analyses due to the small number of DKT ROIs in these networks.

tral (r=0.63), and left caudal-middle-frontal (r=0.62) regions across all software pairs. The pairwise thickness distributions for three randomly selected exemplar ROIs corresponding to different levels of median correlations across software tools are shown in Figure 3. The exemplar ROI comparison suggests that ROIs with high correlation levels tend to have lower overlap between the pairwise thickness distributions.

The covariance matrix of ROIs and subsequently derived structural network metrics reveal several software specific differences. First, the covariance matrix shows large variation of patterns across software tools (see Figure 4-middle). All software tools show strong bilateral symmetry evidenced by the high correlation values on the diagonal representing hemispheric ROI pairs. Interestingly, CIVET features show stronger intra-hemispheric correlation between ROIs compared to the inter-hemispheric values. The DKT ROIs are grouped based on their membership in the Yeo resting state networks [43] to compute graph theoretic metrics. Figure 4 shows the variation in the two commonly used metrics. Figure 4-top shows the impact of correlation threshold, typically used for denoising graph-edges, on the fundamental measure of graph density. The three FS versions show relatively similar performance for all resting state networks, with somatomotor and default mode exhibiting highest and lowest densities, respectively. Compared to FS values, ANTs and CIVET show different magnitudes and/or rankings of graph densities across networks. These differences are further amplified in the graph degree-centrality measurements across networks. Figure 4-bottom shows high intra-network regional variance in degree-centrality for FS versions. This variance is relatively smaller for ANTs and CIVET but these software show largely different magnitudes of centrality, particularly in limbic and default mode networks.
Comparison across QC procedures did not show any substantial impact on correlation values. Feature comparison for a given software tool (e.g. FS6.0) across different parcellations is not trivial due to the lack of correspondence between various parcellation schemes.

Task-free individual (TF-I) analysis. Individual comparisons using thickness measures from DKT parcellation are performed across the five software tools with an identical set of subjects. Commonly used 2-dimensional t-SNE embeddings show strong similarity between subjects for a given software tool (see Figure 5). The three FS versions are much more similar to each other than any FS version is to CIVET or ANTs, reflecting that the different versions of FS share methodological and technical components. Individual covariance is quantified using clustering consistency (CC) that measures the fraction of pairs of individuals assigned to the same cluster with two different feature sets (e.g. ANTs vs. CIVET). Based on CC metric, hierarchical clustering with Euclidean distance similarity and Ward's linkage criterion shows poor stability $(CC\epsilon[0.52, 0.61])$ across software tools and between FS versions (see Table 4). In contrast, hierarchical clustering with correlation metric and average linkage criterion shows highly stable cluster membership

	Controls					ASD				
	ANTs	CIVET	FS5.1	FS5.3	FS6.0	ANTs	CIVET	FS5.1	FS5.3	FS6.0
ANTs	1	0.43	0.45	0.48	0.44	1	0.39	0.39	0.46	0.41
CIVET		1	0.48	0.52	0.52		1	0.44	0.48	0.49
FS5.1			1	0.89	0.84			1	0.87	0.83
FS5.3				1	0.89				1	0.88
FS6.0					1					1

Table 3. Average ROI correlations between software pairs for control and ASD cohorts.

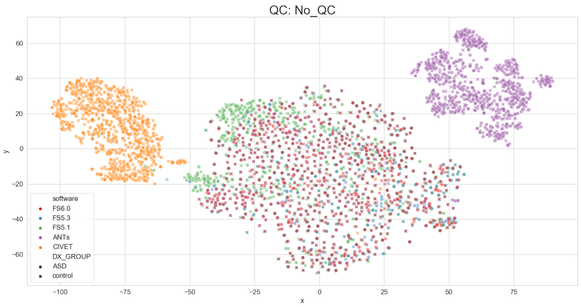

Fig. 5. Task-free individual (TF-I) analysis: Two dimensional t-SNE representation of all individuals (No QC). The colors indicate the software tool used, and the marker style refers to the diagnostic group.

$(CC\epsilon[0.962, 0.997])$.
Comparison across QC procedures did not show any substantial impact on t-SNE representations or clustering consistency values. Individual comparisons across different parcellations for a given software tool (e.g. FS6.0) are not particularly informative due to the lack of correspondence between various parcellation spaces.

Task-driven neurobiological (TD-N) analysis. The mass-univariate regression models per ROI region suggest cortex-wide association between age and thickness values for all software tools, with the exception of the CIVET-based analysis, which excludes bilateral insular regions (see Figure 6). QC procedures seem to have varying impact on the significant regions depending on the software tool. The aggregate ranking suggests higher variation in significant regions for ANTs and CIVET. In contrast the FreeSurfer versions offer relatively similar performance - with consistent exclusion of entorhinal regions. The stringent manual QC sample severely reduces the number of significant regions, which may be due to reduced statistical power.
Parcellation comparisons for FreeSurfer 6.0 reaffirm cortex-wide association between age and thickness values across the three parcellation schemes with some exclusions in medial and superior temporal gyri for Destrieux and STGa, PIR, TGd, TGv, PHA1, EC, PeEc with Glasser (see Figure 7). Lenient QC does not seem to change the distribution of significant regions. However, stringent and automatic QC based results additionally exclude regions from precentral gyri for all three atlases.

Task-driven individual (TD-I) analysis. The RF model based predictions show consistent Root Mean Square Error (RMSE) performance (5.7 - 7.2 years) across software tools, with FS versions showing marginally lower error (see Figure 8). All model performances are statistically significant when compared against a null model. The average RMSE for the control cohort is lower than the ASD cohort; as expected per the null model, however the difference is statistically insignificant. Lenient QC does not have an impact on RMSE distributions. Stringent QC reduces the average RMSE for all software tools (3 - 5 years) and the null model. Automatic QC reduces the average RMSE as well as its variance for all software tools (3.8 - 4.7 years). Interestingly with the automatic QCs (low- and high-dimensional), the null models expectations are reversed as the average RMSE for ASD subjects is now lower than that of controls.
Parcellation-based comparisons show similar RMSE performance despite the differences in granularity of regions and the consequent number of input features to the ML models (see Figure. 9). The RMSE trends with respect to QC are also consistent, with both stringent and automatic QC reducing the average RMSE and the latter yielding a much tighter distribution of error. The null model shows lower expected error for the control cohort compared to the ASD, except for the automatic QC based analyses, where this expectation is reversed.

ROI importance from Random Forest (RF). The cross-validated recursive feature elimination (RFE) procedure yields drastically different feature sets across software tools (see Figure 10). Overall all software tools require a small number of features for age prediction of control subjects (n $\epsilon[3, 20]$) compared to ASD subjects ($n\epsilon[41, 60]$). RFE seems to be very sensitive to the QC procedures as these yield different feature sets with no apparent consistent trends for controls or ASD cohorts. The parcellation comparisons also show varied selection of features. Despite the larger number of regions for Destrieux and Glasser parcellations, the number of predictive features remain relatively small. The sensitivity to QC procedure appears to reflect in the parcellation analysis as evidenced by large spikes in feature counts for both control and ASD cohorts.

Validation analysis. For the HCP dataset, the feature comparisons based on DKT parcellation yielded an average Pearson correlation of 0.66 between CIVET2.1 and FS6.0 (ABIDE: r=0.52). The regions exhibiting low correlations were also consistent with AIBIDE analysis, and comprised cingulate regions, orbitofrontal regions, entorhinal, perical-

	Similarity: Euclidean distance, linkage: Ward's method					Similarity: correlation, linkage: average				
	ANTs	CIVET	FS5.1	FS5.3	FS6.0	ANTs	CIVET	FS5.1	FS5.3	FS6.0
ANTs	0.797	0.5	0.521	0.517	0.522	0.991	0.970	0.962	0.972	0.972
CIVET		0.717	0.5	0.5	0.5		0.994	0.982	0.992	0.992
FS5.1			0.78	0.609	0.529			0.997	0.990	0.985
FS5.3				0.703	0.499				0.997	0.995
FS6.0					0.619					0.997

Table 4. Clustering consistency between software pairs. The diagonal shows expected overlap based on 100 bootstrap samplings of features (31 ROIs) for a given software tool.

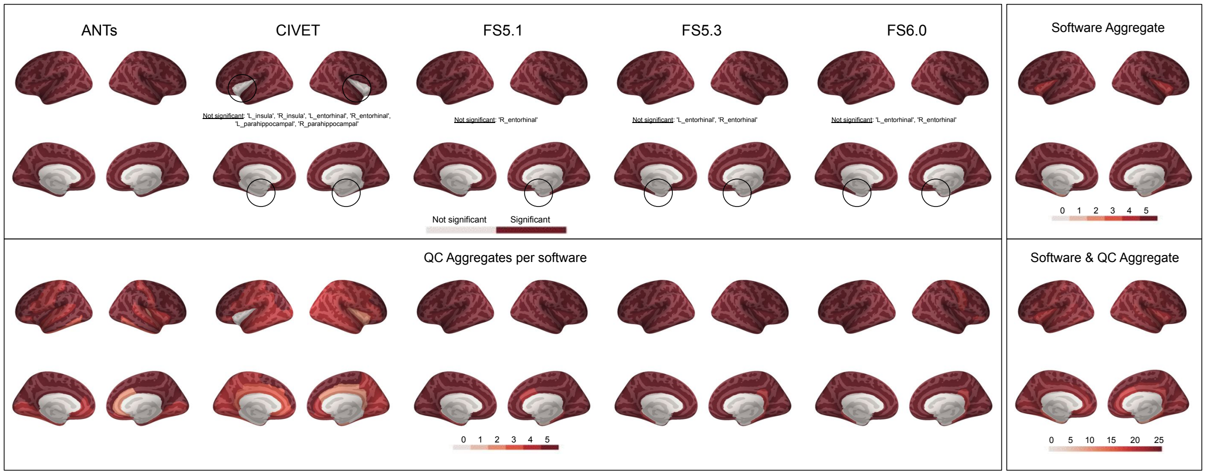

Fig. 6. TD-N analysis: Significant ROI differences with various software and QC levels. Significance levels are corrected for multiple comparisons. Aggregate ranks are assigned based on performance agreement among five software and five QC procedures. Lower rank implies fewer QC procedures yielding the same results.

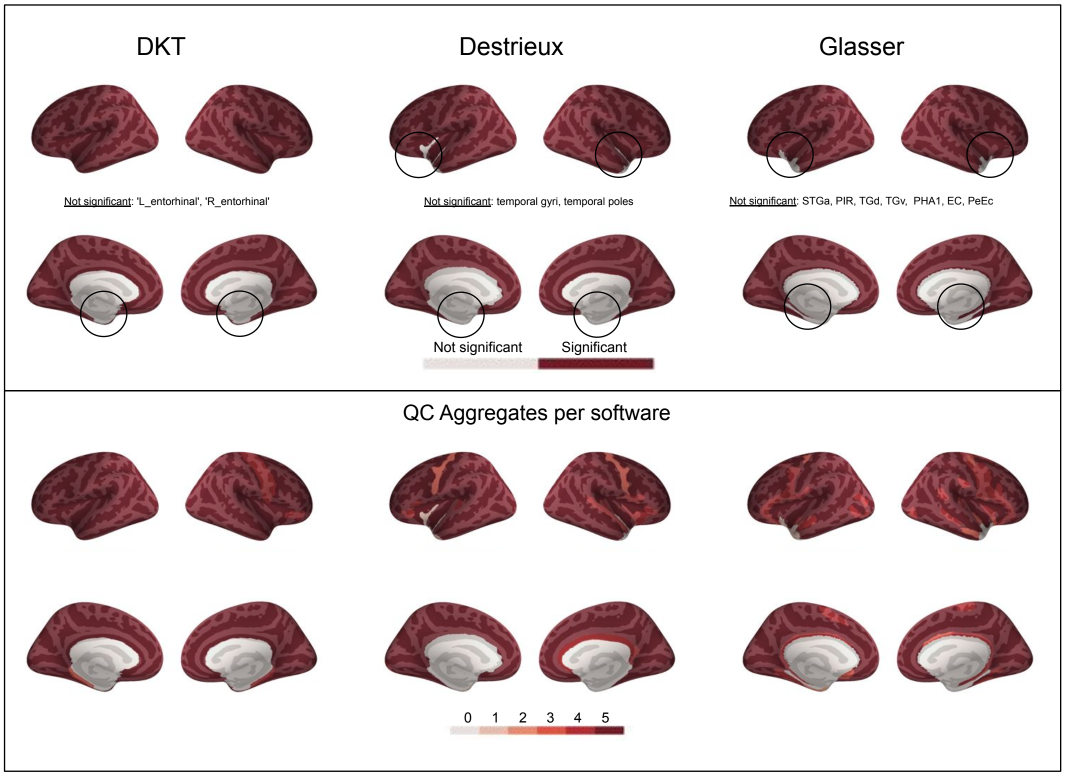

Fig. 7. TD-N analysis: Significant ROI differences with various parcellations and QC levels. Significance levels are corrected for multiple comparisons using the Bonferoni procedure. Aggregate ranks are assigned based on performance agreement between the five QC procedures. Lower rank implies fewer QC procedures yielding the same results.

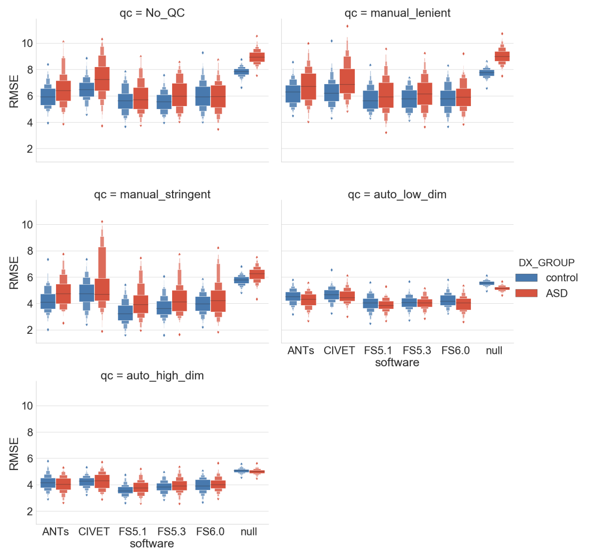

Fig. 8. Task-driven individual (TD-I) analysis: Individual age prediction with various software and QC levels stratified by diagnosis. Performance is cross-validated using a Random Forest model over 100 shuffle-split iterations.

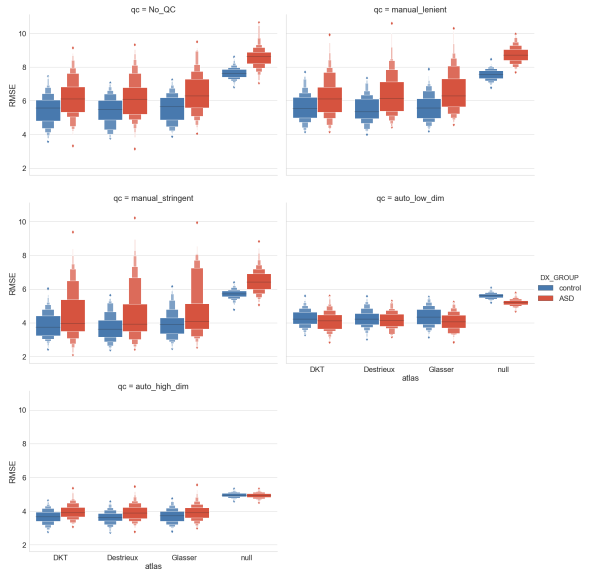

Fig. 9. Task-driven individual (TD-I) analysis: Individual age prediction with various parcellations and QC levels stratified by diagnosis. Performance is cross-validated using a Random Forest model over 100 shuffle-split iterations.

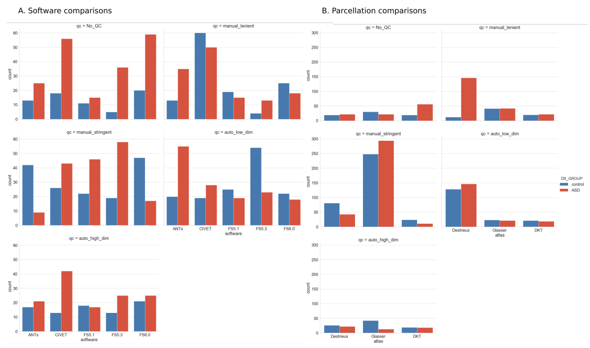

Fig. 10. Predictive feature set count with various (A) software and (B) parcellations for different QC levels stratified by diagnosis. Optimal predictive features are selected using cross-validated recursive feature elimination procedure.

carine, and insula.

Discussion

In this work, we aimed to assess the reproducibility of phenotypic features and subsequent findings subjected to preprocessing pipeline variation along three axes: 1) image processing tool, 2) anatomical priors, 3) quality control. We emphasize that the goal here is not to deliberate specific biological and individual interpretation from the analyses, but rather to highlight the differences among the findings themselves, a key information for the large community of researchers using anatomical brain imaging in their studies.

In the TF-N analysis, we observe a weak ROI-wise correlation across software pairs (see Figure 3). Although software specific biases are expected in biological phenotypic estimates, the level of diminished correlation is striking. One can explain this performance for the comparisons involving ANTs as it is the only software that operates in the voxel (volume) space. However, a similarly poor performance is seen with CIVET and FreeSurfer, both of which operate in a vertex (surface) space for cortical thickness estimation. Since individual ROI-based measures are frequently used in the downstream mass-univariate models, the lack of consensus across software tools is likely to yield different results. Moreover, the varying ROI covariance patterns across the software (see Figure. 4) suggest weak multivariate similarity, which again strongly increases the dependence of findings and biological interpretations on the software choice. For instance, the bilateral symmetry between cortical ROIs may only be inferred with CIVET due to its algorithmic specificities. Lastly, the lack of impact from QC suggests that these effects are systemic and not driven by outliers.

In the TF-I analysis, software tool specific t-SNE similarity is encouraging and expected. The t-SNE embeddings also highlight stronger differences between software tools compared to the differences in diagnostic groups (see Figure 5). This partly explains the high difficulty in training generalizable ML models across studies employing different preprocessing pipelines. The poor clustering consistency with commonly used Ward's linkage criterion is alarming (see Table 4). Given that data-driven clustering is a typical practice to identify subgroups of patients or define meaningful biomarkers [44,45], clustering membership that is highly sensitive to the preprocessing pipeline may go undetected by the stability tests performed on the final set of processed features.

In the TD-N analysis, the software and parcellation comparisons show relatively consistent spatial associations for the age regression models (see Figure 7-8). There are some software-specific regional peculiarities (e.g. insular regions with CIVET), which also behave differently with various QC procedures as can be seen by more variable performance of ANTs and CIVET. These sensitivities should be noted as they could suggest methodological limitations or bias in the software. The overall cortex-wide association of thickness with age is expected as various studies have reported the same in healthy and ASD populations [38,40,46,47]. Direct comparison with other studies is challenging due to differences in the

underlying statistical models, which produce varying topologies of wide-spread associations, and the direction of change in the cortical thickness. The results in this work suggest that the lack of strong ROI (univariate) correlation between a pair of software tools does not impact the task-driven mass-univariate analysis. However, we note that this is highly specific to the task at hand, as well as model selection procedures, which are beyond the scope of this work. We speculate that localized effects are likely to be more sensitive to the univariate pairwise relationships, and therefore a novel biological finding must be reported with high scrutiny to exclude pipeline specificities.

In the TD-I analysis, age prediction with random forest is stable subject to software and parcellation variations (see Figures 8-9). The RMSE performance of 3.8-4.7 years is comparable to the similar previous age prediction studies [16,37,38] that report RMSE in ranges of 6-12 years or mean absolute error of 1.7-1.95 years. The stability of performance could potentially be attributed to the relatively large sample sizes. It is encouraging to see that biological noise does not induce large variations into individual predictions. It is also important to note the impact of QC on the model performance and the null distributions for a given population (i.e. controls vs ASD). These alterations in the expected null performance need to be reported in order to fairly evaluate the improvements offered by a novel model on a given sample. Although random forest seems to be stable for individual predictions, the feature importance assessments by the same model are highly variable (see Figure 10). One explanation for this behaviour could be that in the presence of noisy biological features, ML models assign a relatively flat distribution of importance to the features. Variation in feature sets or sample sizes, as dictated by the selected preprocessing pipeline, would thus yield a drastically different feature ranking in a given iteration of the analysis. This needs to be taken into account if ML models are used to make biological inferences.

The validation analysis with HCP allowed us to replicate our feature correlations findings on an independent dataset. Similar to the ABIDE analysis, HCP data showed consistent low correlation between the ROI thickness values produced by FS6.0 and CIVET2.1. Moreover, there is a large overlap in the regions (i.e. cingulate regions, orbitofrontal regions, entorhinal, and insula) exhibiting the low correlations. This suggests that the low correlations are mainly driven by the algorithmic differences and not by the dataset. The pericalcarine was the exception to this common regional subset, which had a low correlation only in the HCP dataset, possibly due to dataset specific peculiarities. Nevertheless this highlights the need for larger meta-analyses to identify tool-specific and dataset-specific variability in findings.

Limitations. Although in this work we aimed at assessing the impact of pipeline vibration along three different axes, we only considered a subset of permutations in the analysis. This was primarily due to practical reasons such as the lack of availability of common parcellation definitions for all software tools. Therefore we could not compare software tools with Destrieux and Glasser parcellations. We also limited the scope of this work to structural features, and did not consider functional or diffusion measures. With the increasing popularity of sophisticated, derived measures from highly flexible functional preprocessing pipelines with a multitude of design parameters, it is critical to understand and quantify the inherent variability and its impact on downstream findings. We defer this endeavor to future studies and refer to [6] for some progress in this direction.

Conclusions

This work highlights the variability introduced by preprocessing pipelines, which is only a part of the larger issue of reproducibility in computational neuroimaging. We understand that the computational burden of comparative analyses such as described here can be infeasible in many studies. This necessitates undertaking of large meta analytic studies to understand software specific biases for various populations stratified by demographics and pathologies. At the single study level, we encourage the community to process data with different tools as much as possible and report variation of the results. We also propose to systematically report positive and negative results with different parcellations. This will improve confidence levels in the findings and help to better understand the spatial granularity associated with the effect of interest, while facilitating comparisons of common atlases across tools. Lastly, we also recommend assessing the sensitivity of findings against varying degrees of stringency for the QC criteria. Only with wide-spread adoption of rigorous scientific methodology and accessible informatics resources to replicate and compare processing pipelines can we address the compounding problem of reproducibility in the age of large-scale, data-driven computational neuroscience. The availability of containerized and well documented pipelines together with the necessary computing resources will mitigate the variability of results observed and direct the community towards understanding these differences, as well as further develop methodological validation and benchmarking.

ACKNOWLEDGEMENTS

This work was partially funded by National Institutes of Health (NIH) NIH-NIBIB P41 EB019936 (ReproNim) NIH-NIMH R01 MH083320 (CANDIShare) and NIH RF1 MH120021 (NIDM), the National Institute Of Mental Health of the NIH under Award Number R01MH096906 (Neurosynth), as well as the Canada First Research Excellence Fund, awarded to McGill University for the Healthy Brains for Healthy Lives initiative and the Brain Canada Foundation with support from Health Canada. We thank Gleb Bezgin, John Lewis, and David Kennedy's group for compiling manual QC lists used in this work. We are very grateful for a very thorough and insightful review of the manuscript by PJ Toussaint. We also thank Satrajit Ghosh for helping us extend this work as a stand-alone module for vibration analysis in future neuroimaging workflows.

Declarations. *Competing interests*

None of the authors have any financial competing interests with the content of the manuscript.

Supplementary information

Below are the validation results from task-free analyses on the HCP dataset. Figure 11 shows the regional correlations between CIVET2.1 and FS6.0 software. Figure 12 shows the t-SNE plot that highlights the software driven differences on individual clusters.

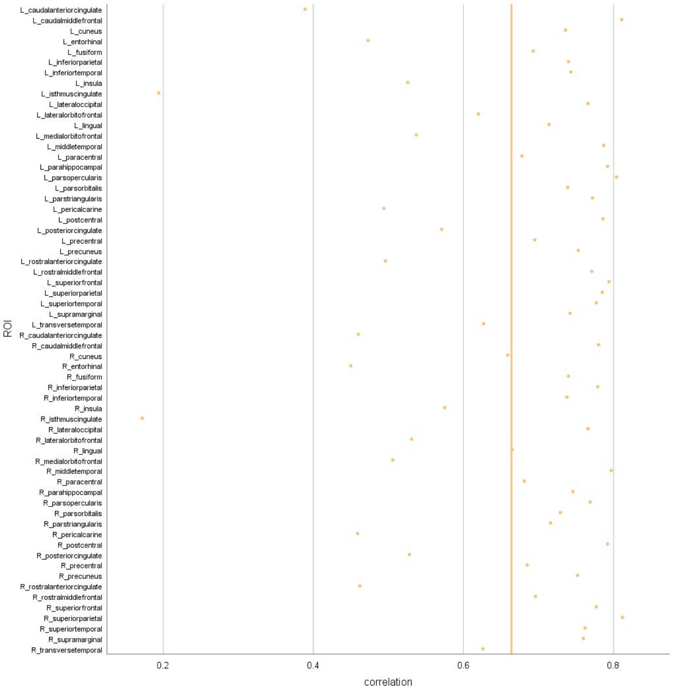

Fig. 11. TF-N analysis for the HCP dataset: Left) Correlation between cortical thickness values for CIVET2.1 and FS6.0 measured independently over ROIs for control and ASD groups. The vertical lines represent the mean correlation across all ROIs, defined using DKT parcellation.

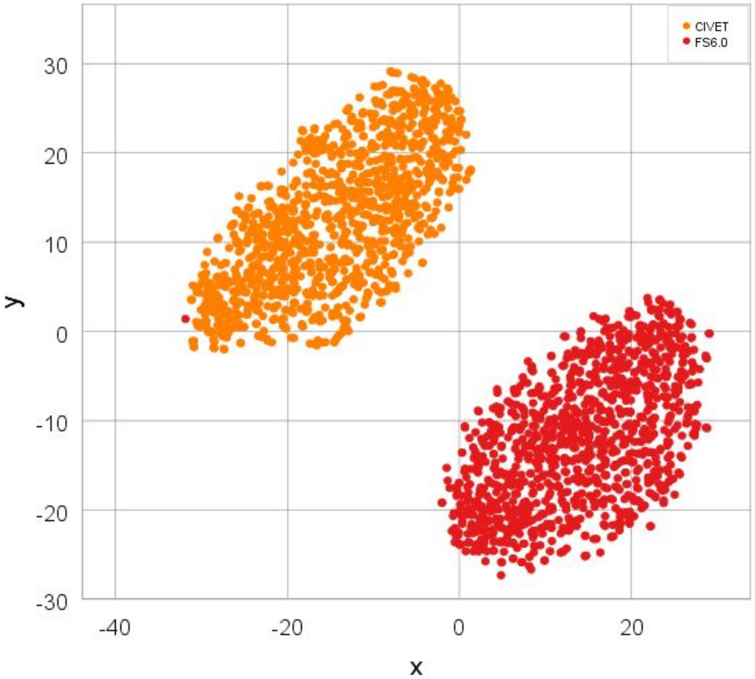

Fig. 12. TF-N analysis for the HCP dataset: Left) Correlation between cortical thickness values for CIVET2.1 and FS6.0 measured independently over ROIs for control and ASD groups. The vertical lines represent the mean correlation across all ROIs, defined using DKT parcellation.

References

1. Milkowski M, Hensel WM, Hohol M. Replicability or reproducibility? On the replication crisis in computational neuroscience and sharing only relevant detail. Journal of Computational Neuroscience. 2018. pp. 163–172. doi:10.1007/s10827-018-0702-z

2. Fanelli D. Opinion: Is science really facing a reproducibility crisis, and do we need it to? Proc Natl Acad Sci U S A. 2018;115: 2628–2631.

3. Baker M. 1,500 scientists lift the lid on reproducibility. Nature. 2016;533: 452–454.

4. Ioannidis JPA. Why most published research findings are false. PLoS Med. 2005;2: e124.

5. Nosek BA, Cohoon J, Kidwell M, Spies JR. Estimating the Reproducibility of Psychological Science. doi:10.31219/osf.io/447b3

6. Bowring A, Maumet C, Nichols TE. Exploring the Impact of Analysis Software on Task fMRI Results. doi:10.1101/285585

7. Carp J. On the plurality of (methodological) worlds: estimating the analytic flexibility of FMRI experiments. Front Neurosci. 2012;6: 149.

8. Button KS, Ioannidis JPA, Mokrysz C, Nosek BA, Flint J, Robinson ESJ, et al. Power failure: why small sample size undermines the reliability of neuroscience. Nat Rev Neurosci. 2013;14: 365–376.

9. Eklund A, Nichols TE, Knutsson H. Cluster failure: Why fMRI inferences for spatial extent have inflated false-positive rates. Proc Natl Acad Sci U S A. 2016;113: 7900–7905.

10. Benjamin DJ, Berger JO, Johannesson M, Nosek BA, Wagenmakers E-J, Berk R, et al. Redefine statistical significance. Nat Hum Behav. 2018;2: 6–10.

11. Lakens D, Adolfi FG, Albers CJ, Anvari F, Apps MAJ, Argamon SE, et al. Justify your alpha. Nature Human Behaviour. 2018;2: 168.

12. Poline J-B. From data sharing to data publishing [version 2; peer review: 2 approved, 1 approved with reservations]. MNI Open Res. 2019;2. doi:10.12688/mniopenres.12772.2

13. Kennedy DN, Abraham SA, Bates JF, Crowley A, Ghosh S, Gillespie T, et al. Everything Matters: The ReproNim Perspective on Reproducible Neuroimaging. Front Neuroinform. 2019;13: 1.

14. Ghosh SS, Poline J-B, Keator DB, Halchenko YO, Thomas AG, Kessler DA, et al. A very simple, re-executable neuroimaging publication. F1000Res. 2017;6: 124.

15. Gorgolewski K, Burns CD, Madison C, Clark D, Halchenko YO, Waskom ML, et al. Nipype: a flexible, lightweight and extensible neuroimaging data processing framework in python. Front Neuroinform. 2011;5: 13.

16. Tustison NJ, Cook PA, Klein A, Song G, Das SR, Duda JT, et al. Large-scale evaluation of ANTs and FreeSurfer cortical thickness measurements. Neuroimage. 2014;99: 166–179.

17. Dickie E, Hodge SM, Craddock RC, Poline J-B, Kennedy DN. Tools Matter: Comparison of Two Surface Analysis Tools Applied to the ABIDE Dataset. Riogrande Odontol. 2017;3: e13726.

18. Di Martino A, Yan C-G, Li Q, Denio E, Castellanos FX, Alaerts K, et al. The autism brain imaging data exchange: towards a large-scale evaluation of the intrinsic brain architecture in autism. Mol Psychiatry. 2014;19: 659–667.

19. Craddock C, Benhajali Y, Chu C, Chouinard F, Evans A, Jakab A, et al. The Neuro Bureau Preprocessing Initiative: open sharing of preprocessed neuroimaging data and derivatives. Front Neuroinform. 2013;7. Available: https://www.frontiersin.org/10.3389/conf.fninf.2013.09.00041/event_abstract

20. Fischl B. FreeSurfer. NeuroImage. 2012. pp. 774–781. doi:10.1016/j.neuroimage.2012.01.021

21. Dale AM, Fischl B, Sereno MI. Cortical Surface-Based Analysis. NeuroImage. 1999. pp. 179–194. doi:10.1006/nimg.1998.0395

22. Collins DL, Louis Collins D, Neelin P, Peters TM, Evans AC. Automatic 3D Intersubject Registration of MR Volumetric Data in Standardized Talairach Space. Journal of Computer Assisted Tomography. 1994. pp. 192–205. doi:10.1097/00004728-199403000-00005

23. Fischl B, Dale AM. Measuring the thickness of the human cerebral cortex from magnetic resonance images. Proceedings of the National Academy of Sciences. 2000. pp. 11050–11055. doi:10.1073/pnas.200033797

24. Imaging Protocols « ENIGMA. [cited 21 Jul 2019]. Available: http://enigma.ini.usc.edu/protocols/imaging-protocols/

25. Compute Canada - Calcul Canada. In: Compute Canada - Calcul Canada [Internet]. [cited 21 Jul 2019]. Available: https://www.computecanada.ca/

26. Sherif T, Rioux P, Rousseau M-E, Kassis N, Beck N, Adalat R, et al. CBRAIN: a web-based, distributed computing platform for collaborative neuroimaging research. Front Neuroinform. 2014;8: 54.

27. Sled JG, Zijdenbos AP, Evans AC. A nonparametric method for automatic correction of intensity nonuniformity in MRI data. IEEE Trans Med Imaging. 1998;17: 87–97.

28. Tustison NJ, Avants BB, Cook PA, Zheng Y, Egan A, Yushkevich PA, et al. N4ITK: improved N3 bias correction. IEEE Trans Med Imaging. 2010;29: 1310–1320.

29. Das SR, Avants BB, Grossman M, Gee JC. Registration based cortical thickness measurement. Neuroimage. 2009;45: 867–879.

30. Klein A, Tourville J. 101 labeled brain images and a consistent human cortical labeling protocol. Front Neurosci. 2012;6: 171.

31. Destrieux C, Fischl B, Dale A, Halgren E. Automatic parcellation of human cortical gyri and sulci using standard anatomical nomenclature. Neuroimage. 2010;53: 1–15.

32. Glasser MF, Coalson TS, Robinson EC, Hacker CD, Harwell J, Yacoub E, et al. A multi-modal parcellation of human cerebral cortex. Nature. 2016;536: 171–178.

33. Desikan RS, Ségonne F, Fischl B, Quinn BT, Dickerson BC, Blacker D, et al. An automated labeling system for subdividing the human cerebral cortex on MRI scans into gyral based regions of interest. Neuroimage. 2006;31: 968–980.

34. Human Connectome Project | Mapping the human brain connectivity. [cited 4 Aug 2019]. Available: http://www.humanconnectomeproject.org/

35. Bezgin G, Lewis JD, Evans AC. Developmental changes of cortical white–gray contrast as predictors of autism diagnosis and severity. Transl Psychiatry. 2018;8: 249.

36. Zhang W, Groen W, Mennes M, Greven C, Buitelaar J, Rommelse N. Revisiting subcortical brain volume correlates of autism in the ABIDE dataset: effects of age and sex. Psychol Med. 2018;48: 654–668.

37. Madan CR, Kensinger EA. Predicting age from cortical structure across the lifespan. Eur J Neurosci. 2018;47: 399–416.

38. Khundrakpam BS, Tohka J, Evans AC, Brain Development Cooperative Group. Prediction of brain maturity based on cortical thickness at different spatial resolutions. Neuroimage. 2015;111: 350–359.

39. Khundrakpam BS, Lewis JD, Kostopoulos P, Carbonell F, Evans AC. Cortical Thickness Abnormalities in Autism Spectrum Disorders Through Late Childhood, Adolescence, and Adulthood: A Large-Scale MRI Study. Cereb Cortex. 2017;27: 1721–1731.

40. Sabuncu MR, Konukoglu E, Alzheimer's Disease Neuroimaging Initiative. Clinical prediction from structural brain MRI scans: a large-scale empirical study. Neuroinformatics. 2015;13: 31–46.

41. Bedford SA, Park MTM, Devenyi GA, Tullo S, Germann J, Patel R, et al. Large-scale analyses of the relationship between sex, age and intelligence quotient heterogeneity and cortical morphometry in autism spectrum disorder. Mol Psychiatry. 2019. doi:10.1038/s41380-019-0420-6

42. Van Essen DC, Smith SM, Barch DM, Behrens TEJ, Yacoub E, Ugurbil K, et al. The WU-Minn Human Connectome Project: an overview. Neuroimage. 2013;80: 62–79.

43. Yeo BTT, Krienen FM, Sepulcre J, Sabuncu MR, Lashkari D, Hollinshead M, et al. The organization of the human cerebral cortex estimated by intrinsic functional connectivity. J Neurophysiol. 2011;106: 1125–1165.

44. Easson AK, Fatima Z, McIntosh AR. Functional connectivity-based subtypes of individuals with and without autism spectrum disorder. Netw Neurosci. 2019;3: 344–362.

45. Hrdlicka M, Dudova I, Beranova I, Lisy J, Belsan T, Neuwirth J, et al. Subtypes of autism by cluster analysis based on structural MRI data. Eur Child Adolesc Psychiatry. 2005;14: 138–144.

46. Sowell ER, Thompson PM, Leonard CM, Welcome SE, Kan E, Toga AW. Longitudinal mapping of cortical thickness and brain growth in normal children. J Neurosci. 2004;24: 8223–8231.

47. Ecker C, Shahidiani A, Feng Y, Daly E, Murphy C, D'Almeida V, et al. The effect of age, diagnosis, and their interaction on vertex-based measures of cortical thickness and surface area in autism spectrum disorder. J Neural Transm. 2014;121: 1157–1170.

Personal Cover Click here to access/download;Personal Cover;gigascience-cover-letter.pdf

Dear editors,

Please find our submission "Understanding the impact of preprocessing pipelines on neuroimaging cortical surface analyses" to be considered for publication in GigaScience.

In this article, we describe the impact of variation of the brain cortical surface preprocessing both on biological results and on prediction results, using two large datasets, the ABIDE (patients with Autism Syndrome Disorder and healthy controls), and the Human Connectome Project dataset. We show in different analyses that processing software have a profound impact on biological conclusions as well as on prediction analyses.

While there are publications on the reproducibility of neuroimaging experiment, the impact we are describing and the in depth analyses performed have not been described previously. We think that these results should prompt the neuroimaging community to re-think how results should be obtained and published. We believe that because of the evidence presented in this article, publications should include replication with several processing tools and analyses. As an analogy, the genetic community has realized that GWAS findings needed to be replicated on at least another cohort to confirm the findings. We demonstrate here that to assure the solidity of neuroimaging results, here on the brain cortical surface of ASD and controls, investigators need to consider replications with processing tools. This is of direct interest to neuroscientists, but also points to a more general issue in the life sciences.

Best regards,

On behalf of the authors,
Jean-Baptiste Poline

Associate Professor, Montreal Neurological Institute, McGill University
