# Supplementary material for: Understanding the impact of preprocessing pipelines on neuroimaging cortical surface analyses
Source: Gigascience. 2021 Jan 22;10(1):giaa155. doi: 10.1093/gigascience/giaa155 (PMC7821710; doi:10.1093/gigascience/giaa155)
Supplement: giaa155_Supplemental_File [file giaa155_supplemental_file.pdf]

Supplementary information

Below are the validation results from task-free analyses on the HCP dataset for CIVET2.1 and FreeSurfer (FS) 6.0 software and DKT parcellation. Figure S1 shows the regional correlations between CIVET2.1 and FS6.0 software. Figure S2 shows the t-SNE plot that highlights the software driven differences on individual embeddings.

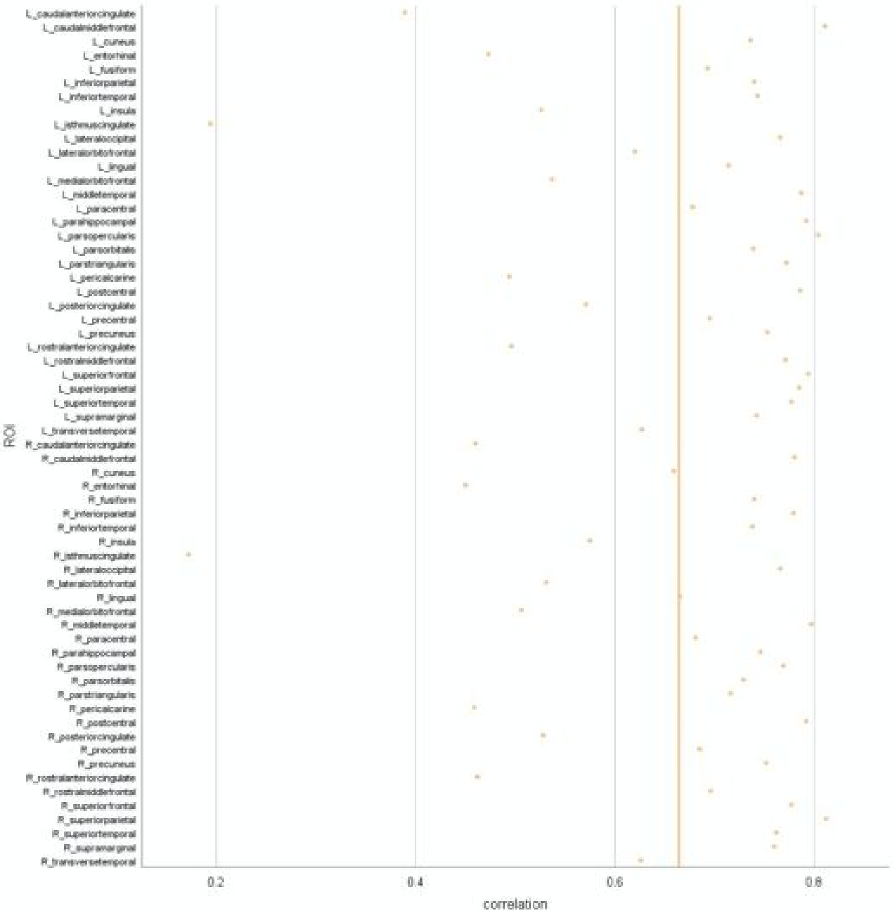

Fig. S1. TF-N analysis for the HCP dataset. Correlation between cortical thickness values for CIVET2.1 and FS6.0 measured independently over ROIs for control and ASD groups. The vertical lines represent the mean correlation across all ROIs, defined using DKT parcellation.

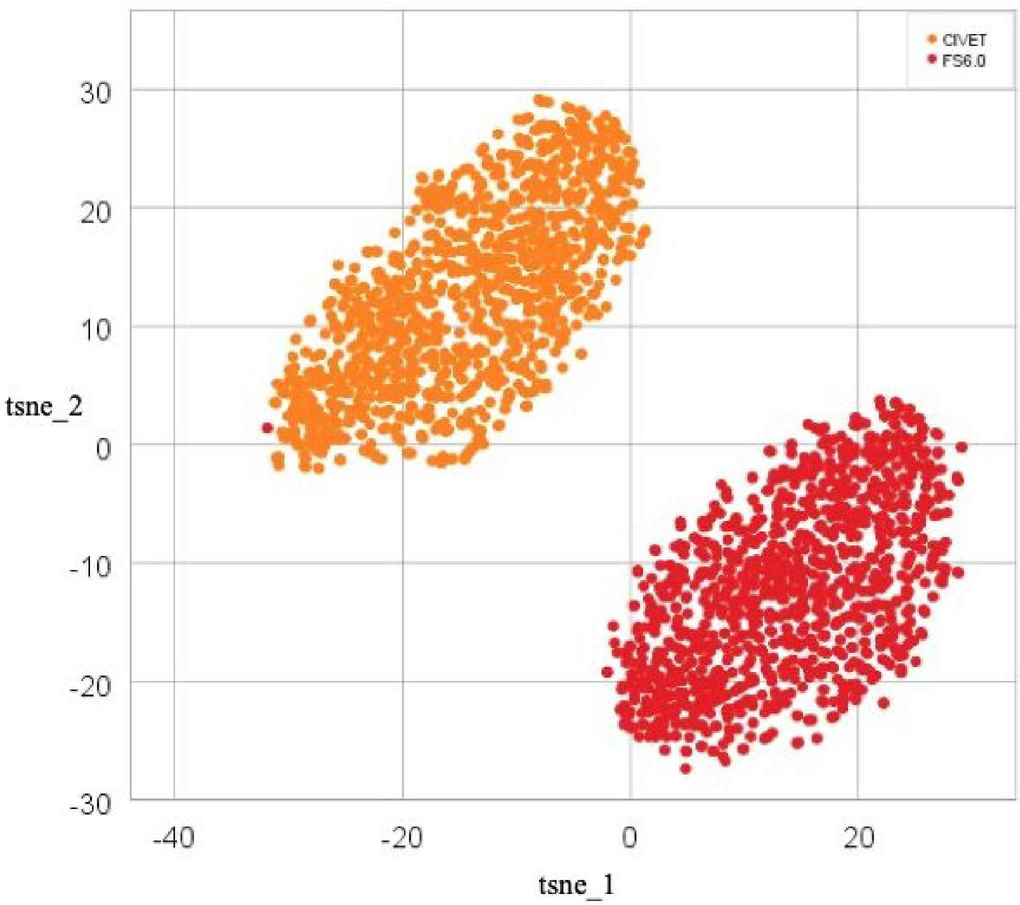

Fig. S2. TF-I analysis for the HCP dataset. t-SNE plot showing difference between individual embeddings for CIVET2.1 and FS6.0 software.
