# Supplementary material for: Understanding the impact of preprocessing pipelines on neuroimaging cortical surface analyses
Source: Gigascience. 2021 Jan 22;10(1):giaa155. doi: 10.1093/gigascience/giaa155 (PMC7821710; doi:10.1093/gigascience/giaa155)
Supplement: giaa155_Response_to_Reviewer_Comments_Original_Submission [file giaa155_response_to_reviewer_comments_original_submission.pdf]

Author's Response To Reviewer Comments

Close

We thanks the reviewers for their feedback. A pdf with the response to reviewers has been attached with the revised manuscript.

Close
