# Supplementary material for: Understanding the impact of preprocessing pipelines on neuroimaging cortical surface analyses
Source: Gigascience. 2021 Jan 22;10(1):giaa155. doi: 10.1093/gigascience/giaa155 (PMC7821710; doi:10.1093/gigascience/giaa155)
Supplement: giaa155_Response_to_Reviewer_Comments_Revision_1 [file giaa155_response_to_reviewer_comments_revision_1.pdf]

Author's Response To Reviewer Comments

Close

Dear Nicole,

I have added the sections required and the gigadb reference. Let me know if you see anything else missing.

All the best,
JB

Close
