# Supplementary material for: Understanding the impact of preprocessing pipelines on neuroimaging cortical surface analyses
Source: Gigascience. 2021 Jan 22;10(1):giaa155. doi: 10.1093/gigascience/giaa155 (PMC7821710; doi:10.1093/gigascience/giaa155)
Supplement: giaa155_Reviewer_1_Report_Original_Submission — Ariel Rokem, PhD -- 8/28/2020 Reviewed [file giaa155_reviewer_1_report_original_submission.pdf]

Reviewer Report

Title: Understanding the impact of preprocessing pipelines on neuroimaging cortical surface analyses

Version: Original Submission **Date:** 8/28/2020

Reviewer name: Ariel Rokem, PhD

Reviewer Comments to Author:

This paper aims to quantify the variability induced by researcher choices in standard analysis of brain imaging data. When conducting this kind of analysis, researchers have many choices, and that could have implications to the inferences and conclusions they make, which the authors describe as "pipeline vibration". Typically, researchers will choose one path and report the results. Here, instead several different choices are made and the differences in downstream inferences are quantified, to disturbing effect.

The authors skillfully use a wide range of analysis methods, and the paper overall paints a rather clear and vivid picture of the issues. Nevertheless, I do have some critiques of the analysis and interpretation. I think that if these were addressed, that would help improve the paper.

My main criticism of the paper is that there are several potential sources of variability that affects results of downstream analyses, but the authors do not attempt to distinguish these. Variability could arise, as assumed here, from systematic differences in the processing. But they could also arise from different sensitivity to noise in the data, or from inherent issues with the measured constructs (e.g., "cortical thickness" and "brain region").

First, a treatment of pipeline vibration across pipelines needs to take into account the robustness of an individual pipeline in face of measurement noise. The comparison across datasets is good, but I would have liked to see a more systematic analysis of test-retest reliability of the pipelines within a dataset. Fortunately, I believe that data for such an analysis is readily available through the Human Connectome Project, which the authors already analyzed. This would help understand whether the variability due to methodological choices made here is similar or much larger than the issues arising from measurement noise. My prediction is that different parts of the analysis will be affected quite differently -- the features selected by RF (which may vary a lot) vis a vis raw cortical thickness numbers (which will be rather stable).

Second, an interesting thing to contemplate here is whether variability between software/parcellation/qc relates to the reality of the underlying constructs examined. While researchers refer to the quantity observed in these studies as "cortical thickness", there is some evidence that what is quantified is much murkier (for an example of this, see https://www.pnas.org/content/116/41/20750). This issue is underscored, for example, by the results presented in Figure 3, where different software (CIVET vs. FS, in particular) seem to make different assumptions about what cortical thickness is, leading to _systematic_ differences. Is there something about processing in the different software that helps makes a systematic decision about how cortical thickness should be quantified? I.e., what is the correct thing to do? Do the results in Figure 4 help us adjudicate this?

Similarly, it is not clear what to make of the reality of the cortical parcellations that are used. Do they impose order where no order is to be found? Is that the cause of the mess observed here? In my opinion, this would be a good point to discuss in the paper that places in such doubt analysis based on these constructs.

Relatedly, I wonder how we should interpret the fact that the error in age prediction with random forests is rather stable across pipelines, while the features selected are rather different? Could this indicate that there is a problem at the feature engineering stage? Would the results of this analysis be more accurate (smaller error) using a more stable feature set? Is there some analysis the authors could do to demonstrate this? For example, combining data across parcels, before submitting to this analysis?

In Figure 5, why is t-SNE used, rather than an easier-to-interpret dimensionality-reduction technique? Is variance in the first few principal components of the data insubstantial? I feel like a dimensionality-reduction technique that uses linear correlations (e.g., PCA) would make the linkage between these results and the results in Figure 3 more readily apparent.

Finally, a clarifying question: I am not sure that I understand the information presented in table 1. Were different subsets of the data submitted to different variations of the pipeline? Why is that? What is the overlap between the subsets? How are correlations computed in Figure 3 if not within ROI across individual? This all seems like pretty central to the analysis approach, so the fact that I don't understand it is worrisome.

Minor comments:

I find the nomenclature used by the authors to describe the different comparisons confusing. In particular, "task free" and "task driven" get confused in my mind with task-based fMRI. Why not "supervised" and "unsupervised"?

As they are, most of the Figures are very hard to read. The data points are all too small to see, and the fonts are uniformly way too small to read. I might just be getting old and cranky, but it hurts my eyes.

Typo: "ABIDE" is mis-spelled as "ABIDIE" on page 7 lower-right.

Methods

Are the methods appropriate to the aims of the study, are they well described, and are necessary controls included? Choose an item.

Conclusions

Are the conclusions adequately supported by the data shown? Choose an item.

Reporting Standards

Does the manuscript adhere to the journal's guidelines on minimum standards of reporting? Choose an item.

Choose an item.

Statistics

Are you able to assess all statistics in the manuscript, including the appropriateness of statistical tests used? Choose an item.

Quality of Written English

Please indicate the quality of language in the manuscript: Choose an item.

Declaration of Competing Interests

Please complete a declaration of competing interests, considering the following questions:

- Have you in the past five years received reimbursements, fees, funding, or salary from an organisation that may in any way gain or lose financially from the publication of this manuscript, either now or in the future?
- Do you hold any stocks or shares in an organisation that may in any way gain or lose financially from the publication of this manuscript, either now or in the future?
- Do you hold or are you currently applying for any patents relating to the content of the manuscript?
- Have you received reimbursements, fees, funding, or salary from an organization that holds or has applied for patents relating to the content of the manuscript?
- Do you have any other financial competing interests?
- Do you have any non-financial competing interests in relation to this paper?

If you can answer no to all of the above, write 'I declare that I have no competing interests' below. If your reply is yes to any, please give details below.

I declare that I have no competing interests

I agree to the open peer review policy of the journal. I understand that my name will be included on my report to the authors and, if the manuscript is accepted for publication, my named report including any attachments I upload will be posted on the website along with the authors' responses. I agree for my report to be made available under an Open Access Creative Commons CC-BY license (http://creativecommons.org/licenses/by/4.0/). I understand that any comments which I do not wish to be included in my named report can be included as confidential comments to the editors, which will not be published.

Choose an item.

To further support our reviewers, we have joined with Publons, where you can gain additional credit to further highlight your hard work (see: https://publons.com/journal/530/gigascience). On publication of this paper, your review will be automatically added to Publons, you can then choose whether or not to claim your Publons credit. I understand this statement.

Yes Choose an item.
